# Shear behavior and mechanical model of novel truss-type steel-reinforced recycled concrete short beam

**DOI:** 10.1038/s41598-026-55035-5

**Published:** 2026-05-29

**Authors:** Chunhui Yang, Siliu Yu, Jinfu Liang

**Affiliations:** 1 School of Civil Engineering, Guangxi Polytechnic of Construction, Nanning, 530007 PR China; 2 MOE Key Laboratory of Earthquake Resistance, Earthquake Mitigation and Structural Safety, Guangzhou University, Guangzhou, 510006 PR China

**Keywords:** Truss-type steel-reinforced concrete, Recycled aggregate concrete, Shear capacity, Short beam, Mechanical model, Engineering, Materials science

## Abstract

This study presents an experimental and theoretical investigation on the shear behavior of a novel truss-type steel-reinforced recycled concrete short beam (TSRSB). Five TSRSB specimens with varying recycled coarse aggregate (RCA) replacement ratios (*r =* 0%, 50%, 100%) and shear span-to-depth ratios (*λ =* 0.76, 1.14, 1.52) were tested under concentrated loading to analyze their failure modes, load-displacement responses, and strain developments. The results indicate that reducing the shear span-to-depth ratio significantly enhances the shear capacity and initial stiffness; for instance, the ultimate load of the specimen with *λ =* 0.76 was 70.1% higher than that with *λ =* 1.52. In contrast, the RCA replacement ratio had a negligible impact on the ultimate load, with a maximum reduction of only 3.2%. A sophisticated finite element model was developed and validated against the experimental results, demonstrating high accuracy with a mean ratio of simulated-to-experimental ultimate load of 1.07. Based on the identified “truss-arch” mechanical model, a practical formula for predicting the shear capacity of TSRSB is proposed. This formula incorporates the influence of the RCA replacement ratio via a reduction factor (*α =* 20/(20 + *r*)) and superposes the contributions of the recycled concrete, stirrups, vertical and diagonal web members of the truss, and the steel flanges. Within the tested parameter ranges (*r* = 0 ~ 100%, λ = 0.52 ~ 1.14), the proposed formula shows good agreement with the experimental data, yielding a mean calculated-to-experimental shear capacity ratio of 1.00 with a coefficient of variation of 0.01. Therefore, the formula can provide a preliminary reference for the shear capacity calculation of specimens falling within these parameter ranges.

## Introduction

The construction industry stands at a critical juncture, facing the dual challenges of environmental sustainability and the ever-growing demand for infrastructure development. A significant part of this challenge is the enormous consumption of natural resources, particularly river sand and natural coarse aggregates for concrete production, and the simultaneous generation of colossal amounts of construction and demolition waste (CDW) worldwide^1,2^. In this context, the utilization of recycled coarse aggregates (RCA) from CDW to produce recycled aggregate concrete (RAC) has emerged as a promising strategy for promoting a circular economy in construction^3,4^. However, the inherent weaknesses of RAC, including lower density, higher water absorption, and the presence of old mortar attached to the original aggregates, often result in inferior mechanical properties compared to natural aggregate concrete, such as reduced compressive strength, tensile strength, and modulus of elasticity^5^. These limitations have hindered the widespread application of RAC in primary structural members, necessitating innovative solutions to overcome its deficiencies.

Meanwhile, steel-reinforced concrete structures, which integrate structural steel sections with concrete, have been widely adopted for their excellent load-bearing capacity, ductility, and construction efficiency^6–9^. The composite action between steel and concrete allows each material to compensate for the weaknesses of the other: the concrete provides restraint against local buckling of the steel section, while the steel enhances the tensile strength and ductility of the composite member^10^. Conventional steel-reinforced concrete members typically use solid web steel sections (e.g., I-sections). While effective, these sections can be heavy, costly, and present challenges in concrete placement and bonding with the surrounding concrete. To address these issues, truss-type steel skeletons have been proposed as an alternative^11^. These skeletons, often fabricated by welding steel bars or small sections into a truss configuration, offer advantages such as lighter weight, easier concrete casting, and improved bond characteristics due to their open web system. More importantly, the diagonal web members in the truss can directly resist shear forces, providing a clear and efficient load path.

The shear behavior of structural members represents a classic yet perpetually critical research topic, particularly for short beams where shear-dominated failures are prevalent^12–14^. The shear capacity of such members is governed by a complex interplay of mechanisms including arch action, dowel action, aggregate interlock, and the contribution of transverse reinforcement^15,16^. Mei et al.^17^ emphasized that for short beams with a shear span-to-depth ratio typically less than 5, arch action becomes particularly significant, leading to a distinct failure mechanism compared to slender beams.

When considering truss-type steel-reinforced recycled concrete short beam (TSRSB), the shear behavior becomes substantially more complex due to the interaction between mechanically distinct RAC and the strategically placed truss-type steel skeleton. Mohammed et al.^18^ demonstrated that RAC exhibits different fracture properties and aggregate interlock capabilities compared to conventional concrete. Meanwhile, He et al.^19^ established that truss-type steel skeletons introduce a distinct internal truss mechanism that works alongside the concrete arch action. Extensive research has been conducted on the shear performance of various composite members. For RAC members, studies by Magbool et al.^20^ and Shoaib et al.^21^ showed that shear capacity generally decreases with increasing RCA replacement ratio, though this reduction can be mitigated by transverse reinforcement confinement. For steel-reinforced concrete members, numerous models exist based on either the superposition principle^22–24^ or strut-and-tie models^25–27^ for discontinuity regions. Research on truss-type steel-reinforced concrete members remains relatively limited but growing. Liu et al.^28^ demonstrated their superior shear resistance and ductility compared to conventional reinforced concrete members. However, existing design guidelines and analytical models, particularly those proposed by Deng et al.^29^, were originally developed for conventional steel–concrete composites. Their applicability to TSRSB with recycled aggregate concrete (RAC) requires further investigation, especially regarding the compressive softening behavior of RAC and the interface interaction between RAC and steel. Moreover, most existing models are empirically based; a more comprehensive mechanical model that accounts for the specific characteristics of this composite system remains to be developed. The truss-arch model effectively combines the arch action predominant in deep or short beams with the truss mechanism provided by the shaped steel and reinforcement. This combined action is particularly suitable for analyzing the shear behavior of short beams with low shear span-to-depth ratios. Therefore, it serves as a rational theoretical foundation for modeling the composite action in the proposed TSRSB system.

This research makes three primary contributions to the field of composite structures: (1) It presents a systematic experimental investigation into the shear behavior of novel TSRSB under concentrated loading, with a particular focus on quantifying the effects of key parameters, namely RCA replacement ratio (*r*) and the shear span-to-depth ratio (*λ*). (2) A sophisticated finite element (FE) model is developed using ABAQUS and rigorously validated against experimental data; this model incorporates advanced constitutive relationships for both RAC and steel components to accurately simulate the complex nonlinear response of the beams. (3) Based on the experimental and numerical findings, a practical mechanical model is established, leading to the derivation of a precise design formula for predicting the shear capacity of TSRSB. This formula uniquely and explicitly integrates the individual contributions of the RAC, stirrups, vertical web members, diagonal web members, and steel flanges, providing structural engineers with a reliable and readily applicable calculation tool that has been validated against experimental results. The findings of this research will provide deep insights into the shear mechanisms of TSRSB and deliver a reliable analytical tool for their design, facilitating the safer and more efficient use of sustainable recycled materials in advanced composite structures.

## Test overview

### Test design

**Fig. 1:**
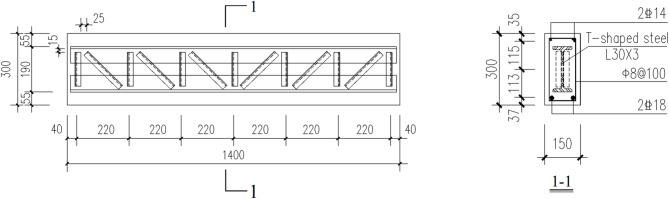
Reinforcement layout.

**Fig. 2:**
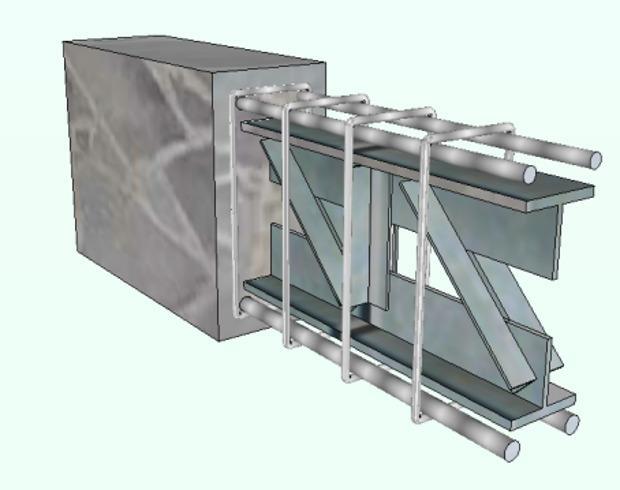
Specimens model.

**Fig. 3:**
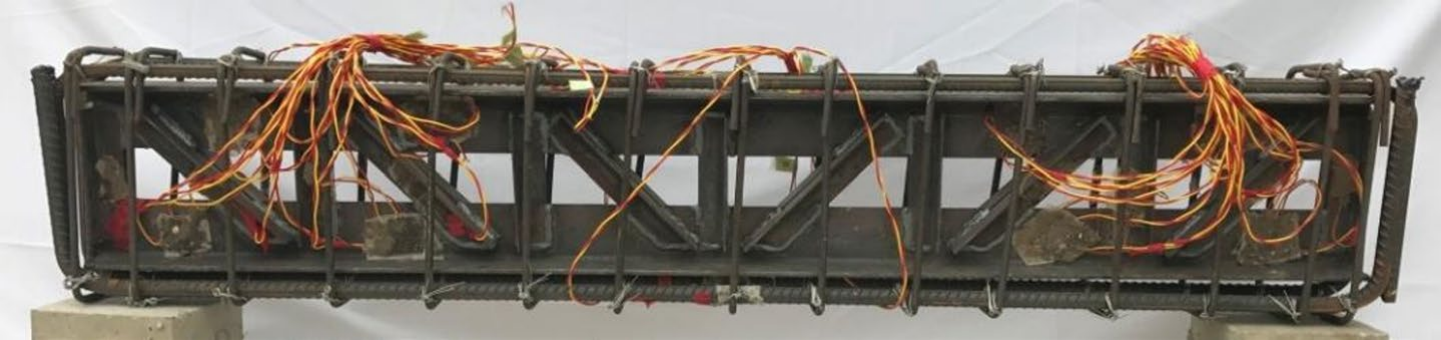
Truss-type steel frame.

In this experimental study, the effects of different recycled aggregate replacement rates (0%, 50%, 100%) and different shear span-to-depth ratios (0.76, 1.14, 1.52) on structural performance were investigated. The design work adhered to national building codes JGJ138-2016^30^ and YB9082-2006^31^, while incorporating the design principles of steel-concrete composite beams. To gain deeper insights into the material’s mechanical behavior, the beam cross-section underwent meticulous optimization to ensure experimental data accurately reflected the material’s mechanical response and intrinsic properties. Five TSRSBs subjected to concentrated loads were fabricated for this study. Their dimensions were span 1400 mm, clear span 1200 mm, and cross-Sect. 150 mm×300 mm, designated as TSRSB-1 to TSRSB-5. Relevant design parameters are listed in Table 1, while the specific reinforcement layout and model structure are shown in Figs. 1 and 2, respectively. During fabrication of the truss steel skeleton, standard I-beam I14 was first cut into symmetrical T-shaped components using oxy-fuel cutting. These components formed the flanges of the skeleton. Subsequently, 30 mm×3 mm equal-angle steel was welded at 45-degree angles to create vertical and diagonal chords, constructing a stable truss structure. Detailed construction is shown in Fig. 3.

**Table 1:** Specimen design.

Specimen number	Replacement rate *r* / %	shear span-to-depth ratio λ	Steel frame form	Shear span a / mm	Concrete strength
TSRSB-1	0	1.14	truss type	300	C40
TSRSB-2	50	1.14	truss type	300	C40
TSRSB-3	100	0.76	truss type	200	C40
TSRSB-4	100	1.14	truss type	300	C40
TSRSB-5	100	1.52	truss type	400	C40

### Material properties

The steel components of the test specimen were fabricated using Q235B structural steel. The longitudinal reinforcement consisted of HRB400 (deformed bar with a yield strength of 400 MPa) bars, while the stirrups were made of HPB300 (plain round bar with a yield strength of 300 MPa) bars, precisely spaced at 100 mm intervals. The structural steel and the reinforcing bars strength was tested according to Chinese standard GB/T 228.1–2021^32^. The measured mechanical properties of the steel are detailed in Table 2. Concrete of strength grade C40 (40 MPa cube strength) was adopted in the design. Natural coarse aggregate (NCA) comprised crushed stone with a continuous gradation, with particle sizes ranging from 5 mm to 20 mm. Recycled coarse aggregate (RCA) was sourced from waste concrete test blocks, processed through manual crushing and rigorous sun-drying to ensure quality. The obtained water absorption was 3.59%, and the apparent density was 2256 kg/m³. The particle size and gradation of these recycled aggregates were comparable to natural coarse aggregates, providing a stable framework for the concrete. For fine aggregates, high-quality natural river sand (medium sand) was selected, while ordinary Portland cement with a strength class of 42.5 MPa and high early strength was used (designated as 42.5R in Chinese standard). During concrete mixing, tap water was employed as mixing water, and the water-cement ratio was strictly controlled to remain within 0.57. The sand ratio is set at 36% to guarantee concrete quality and performance. The concrete strength was tested according to Chinese standard GB/T 50,081 − 2019^33^, compressive strength tests were conducted using an RMT-201 geotechnical and concrete mechanical testing machine. Detailed mix proportions and mechanical performance indicators for the recycled concrete are presented in Table 3.

**Table 2:** Mechanical properties of steel materials.

Steel type	f_y_ / MPa	f_st_ / MPa	E_s_ / MPa
I14	282.7	432.8	2.08 × 10^5^
L30 × 3	303.9	471.2	2.08 × 10^5^
D8 (HPB300)	393.4	563.1	2.14 × 10^5^
D14(HRB400)	432.1	639.2	2.07 × 10^5^
D18 (HRB400)	411.3	617.5	2.06 × 10^5^

Note: *f*_*y*_ denotes the yield strength of steel; *f*_*st*_ denotes the ultimate strength of steel; *E*_*s*_ denotes the elastic modulus of steel.

**Table 3:** The mix ratio of RAC.

*r*/%	Sand ratio/%	Material Consumption/(kg·m^− 3^)					Mechanical performance index/(MPa)			
		Cement	Water	Sand	NCA	RCA	f_cu_	f_c_	f_t_	Ec
0	36	377	215	651	1157	0	43	30.0	2.76	3.38 × 10^4^
50	36	377	215	651	578.5	578.5	40	26.4	2.63	3.29 × 10^4^
100	36	377	215	651	0	1157	41	27.6	2.69	3.33 × 10^4^

Note: *r* denotes replacement ratio of recycled coarse aggregate; *f*_cu_ denotes cube compressive strength of concrete; *f*_c_ denotes axial compressive strength of concrete; *f*_t_ denotes axial tensile strength of concrete; *E*c denotes elastic modulus of concrete.

### Loading and testing plan

**Fig. 4:**
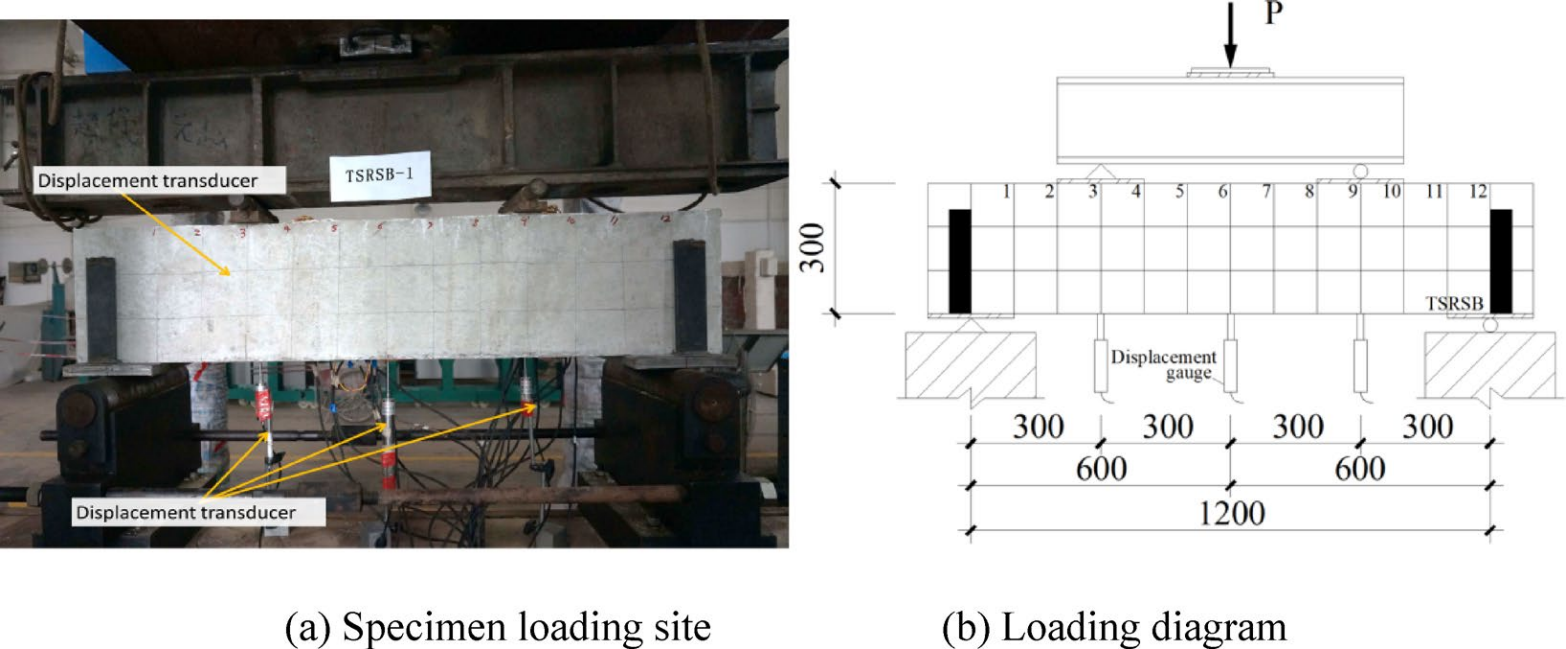
Specimens loading.

**Fig. 5:**
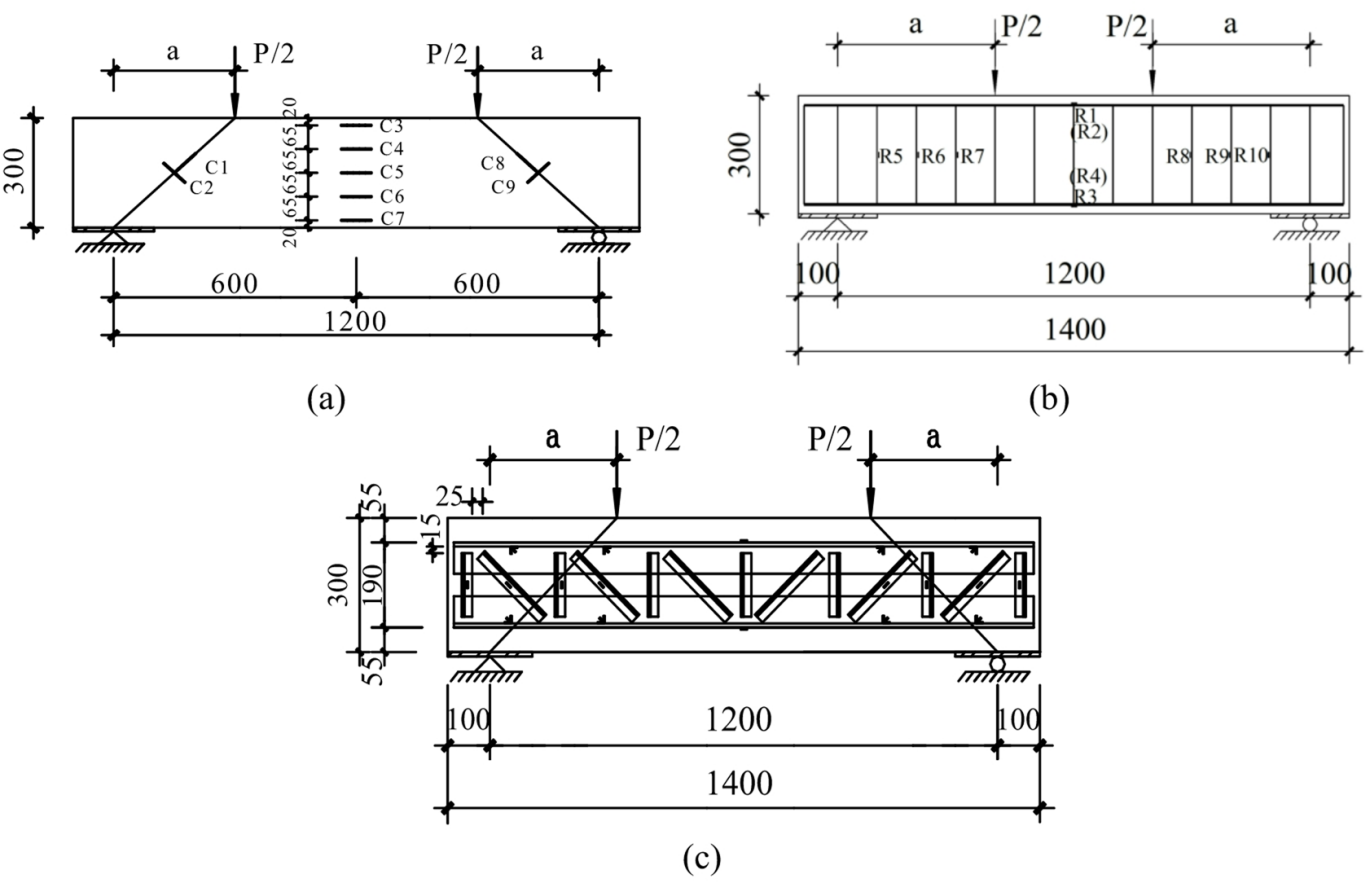
Strain gauge layout diagram for test specimens. (**a**) The arrangement of strain gauges on the concrete surface, (**b**) Layout of strain gauges in the steel bar frame (a=200 mm: R5, R10; a=300 mm, R5, R6, R9, R10; a=400 mm, R5, R6, R7, R8, R9, R10), (**c**) Truss-type steel strain gauge layout (Note: a is 200 mm, 300 mm, 400 mm).

To ensure the accuracy and safety of the test, a simulated loading was performed on the specimen prior to the formal loading phase. The loading applied during this step was only 5% of the estimated maximum load capacity. After confirming all parameters were normal, the preload was unloaded. During the formal loading phase, a force-displacement composite control strategy was employed. Specifically, force control mode was initially employed, with force increments set at 50 kN intervals until approaching 80% of the estimated maximum load capacity. Upon nearing this threshold, the control mode was switched to displacement control, continuously applying force at a rate of 0.5 mm per minute. The test was terminated once significant deformation occurred in the specimen’s mid-section or structural damage resulted from the propagation of oblique cracks in the concrete. The entire loading process is illustrated in Fig. 4. A YE-10,000 F electro-hydraulic servo testing machine was employed to collect real-time load data. This data served not only to evaluate the vertical flexural crack initiation load and diagonal crack initiation load of the specimens but also to determine their ultimate failure load. Based on these measurements, load-deformation curves were plotted for different measurement points to comprehensively analyze the specimens’ mechanical properties. Additionally, strain gauges were arranged on the specimen according to test requirements to monitor strain changes during loading. The strain gauge layout is shown in Fig. 5.

## Test results and analysis

### Failure mode and characteristics of the specimen

#### Failure mode of the specimen

**Fig. 6:**
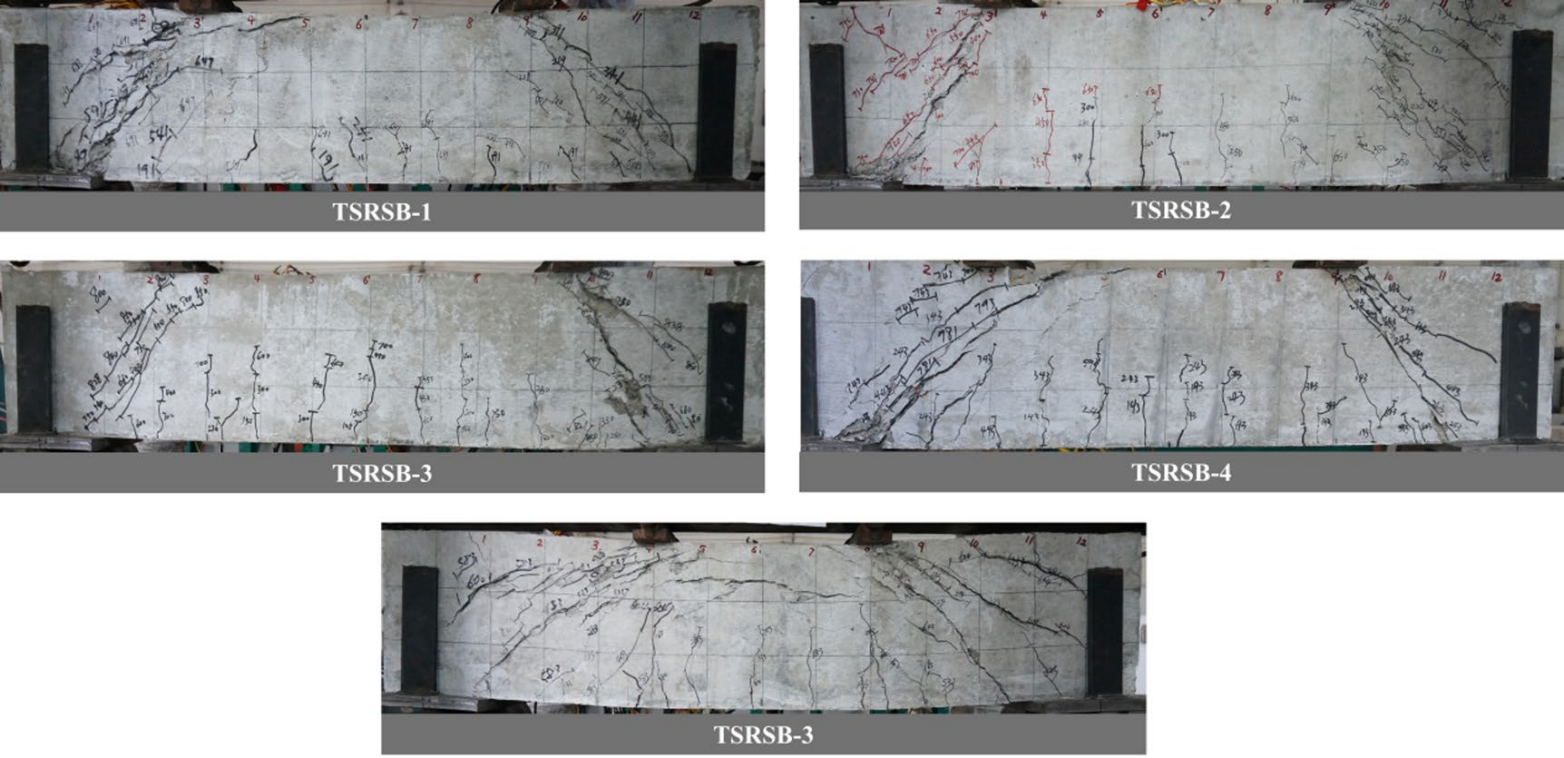
Failure modes of TSRSBs.

The performance parameters and failure modes of TSRSBs are shown in Table 4, while the specimen failure modes are illustrated in Fig. 6. Specimens TSRSB-1 to TSRSB-4 exhibited primary diagonal cracks and diagonal compression member failure in the shear span region, while uniform bending cracks appeared in the pure bending region. TSRSB-5 exhibited primary cracks and diagonal compression failure in the shear span region, with horizontal cracks appearing on the upper flange of the T-shaped steel extending into the pure bending region. Bending cracks were also observed at the bottom of the mid-span. The failure of these specimens was related to the shear span-to-depth ratio and the stiffness of the steel web. With a low shear span-to-depth ratio, forces were primarily transmitted through the shear span section, leading to worsened loading conditions in this region while the pure bending section experienced lighter loads. The steel web resisted loads and constrained the concrete; however, as loads increased, the steel yielded, reducing concrete confinement and exacerbating deformation, ultimately resulting in concrete crushing failure in the diagonal compression zone. The crack progression followed a consistent sequence: initial bending-controlled microcracks emerged at approximately 12–20% of the ultimate load, evolved into a combined bending-shear crack system, and ultimately transitioned to a shear-dominated failure mechanism characterized by the formation, interconnection, and final crushing of diagonal concrete struts near peak load.

**Table 4:** Test results of short beam specimens.

Specimen number	Vertical crack opening Load/kN	Diagonal crack opening Load/kN	Ultimate load/kN	Failure mode
TSRSB-1	121.0	219.0	820.0	Shear oblique compression failure
TSRSB-2	105.0	200.0	794.0	Shear oblique compression failure
TSRSB-3	202.0	187.0	1060.0	Shear oblique compression failure
TSRSB-4	103.0	223.0	804.0	Shear oblique compression failure
TSRSB-5	73.0	188.0	623.0	Shear-bond failure

**Fig. 7:**
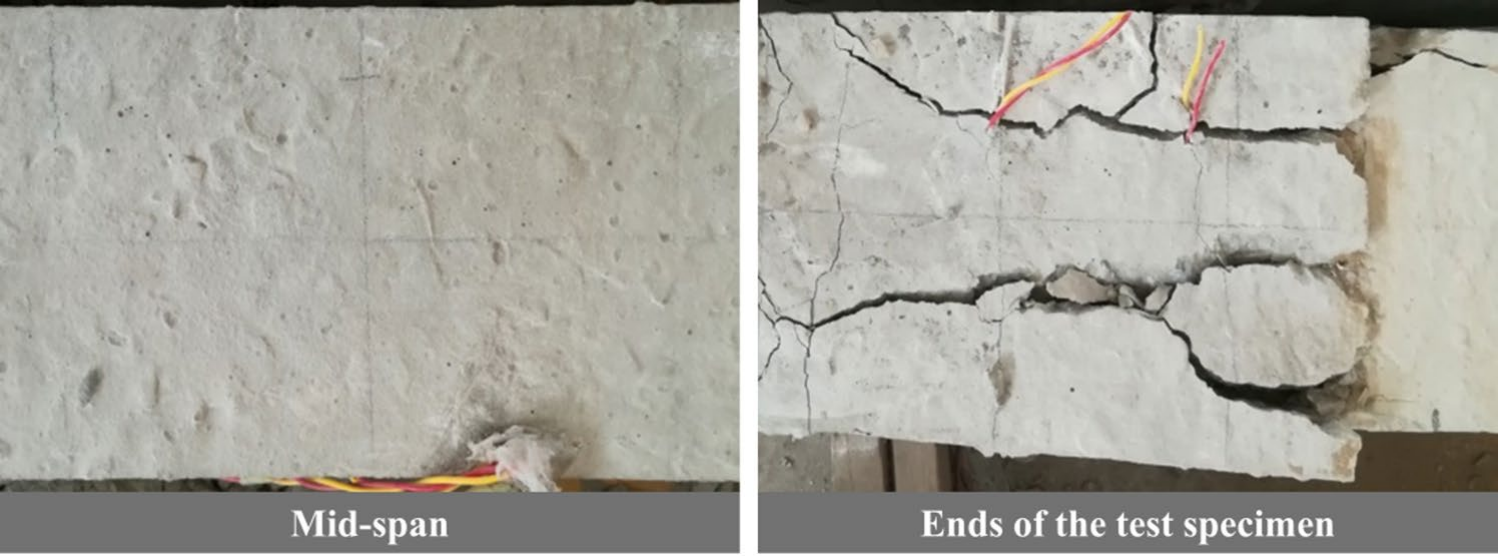
Distribution pattern of TSRSB-1 ~ TSRSB-4 top crack.

**Fig. 8:**
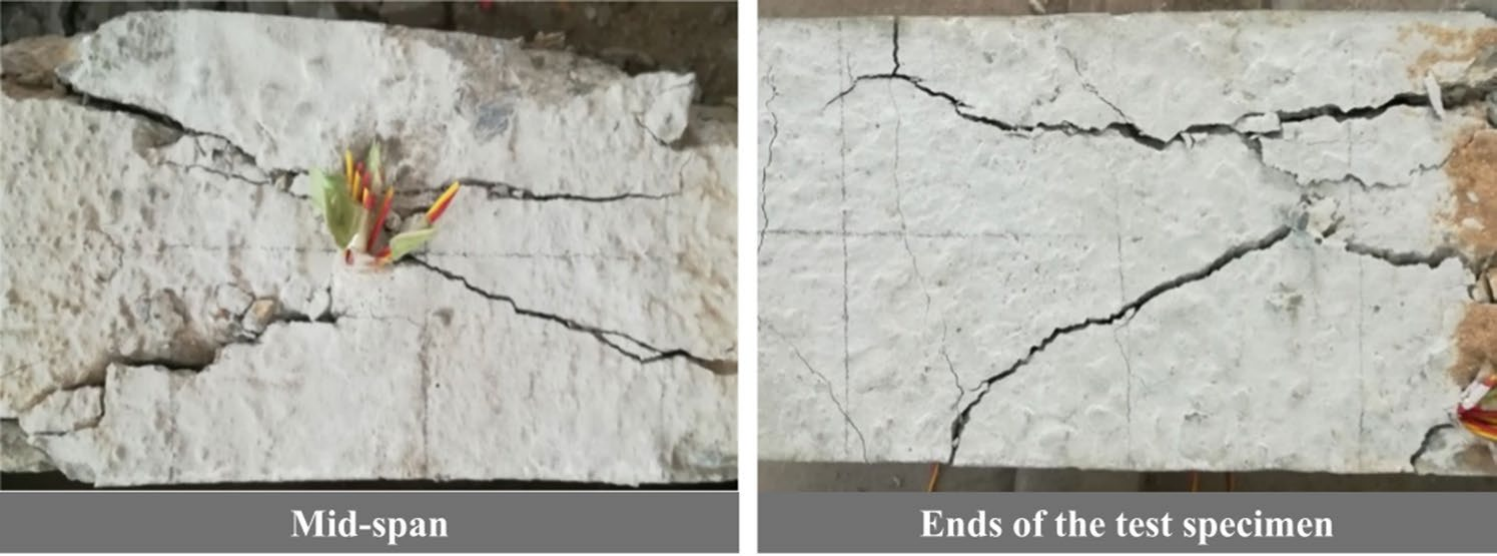
Distribution pattern of TSRSB-5 top crack.

The top failure mode of the TSRSB specimens is shown in Fig. 7. Specimens TSRSB-1 through TSRSB-4 remained undamaged at the mid-span, while splayed cracks appeared outside the loading points. The width of these beams increased after failure. This is attributed to the relatively low bending moment in the pure bending zone, which allowed the load to be transmitted to the supports through diagonal struts. Consequently, the upper part of the pure bending segment did not experience excessive compressive stress and did not reach the concrete’s compressive limit. In the shear span region, influenced by principal tensile stresses, cracks initiated near the loading points and progressively propagated outward, developing into a splayed pattern. Specimen TSRSB-5 exhibited a similar failure pattern at its ends. However, an X-shaped crack pattern formed at its mid-span. This pattern originated from bond slip between the steel section and the surrounding concrete. This slip phenomenon gradually propagated from the shear span into the pure bending zone, accompanied by a reduction in force transfer. Therefore, while superficially appearing as an X-shaped crack, it actually results from the convergence of two sets of splayed cracks in a specific region, as schematically shown in Fig. 8.

**Fig. 9:**
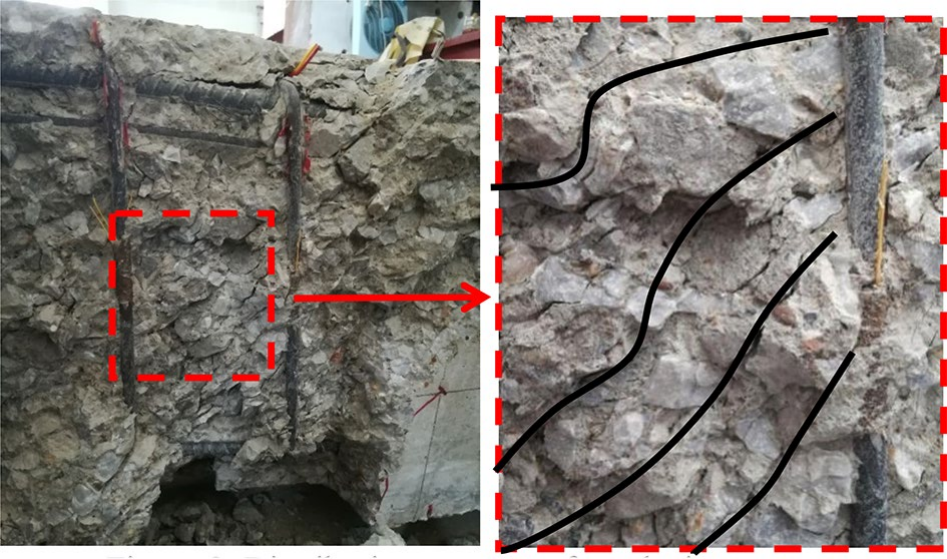
Distribution patterns of cracks in concrete.

**Fig. 10:**
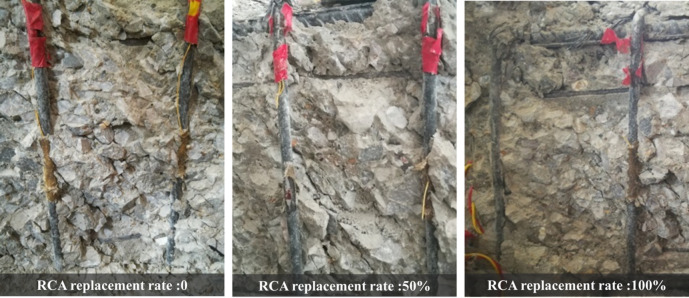
Destruction surface.

**Fig. 11:**
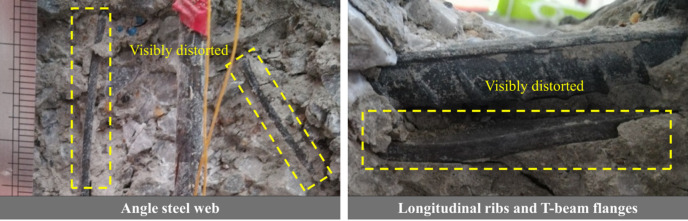
Deformation of longitudinal reinforcement and structural steel.

The failure mode of the shear span section in the TSRSB is shown in Fig. 9. The internal concrete exhibits multiple diagonal cracks supported by the steel skeleton, parallel to the line connecting the loading point to the support, verifying its diagonal compression member structural characteristics. Figure 10 displays failure patterns of specimens with varying RCA replacement rates. As replacement ratios increase, the failure surfaces of recycled concrete specimens become rougher with more cracks, attributed to microcracks in recycled coarse aggregates and the complexity of raw materials. Figure 11 shows significant deformation in the longitudinal reinforcement, upper flange of the T-shaped steel, and web of the angle steel when subjected to shear forces. Strain analysis indicates these components yield before reaching the member’s ultimate load capacity. Although the stirrups appear undistorted, strain analysis also reveals their yield occurs prior to the peak load.

#### Failure characteristics of specimens

In shear performance tests, specimens TSRSB-1 to TSRSB-4 exhibited significant shear-tension failure, while TSRSB-5 demonstrated a unique shear-bond failure mode. The failure process of recycled steel-reinforced concrete short beams can be subdivided into elastic, elastic-plastic, and failure stages. Although these stages share commonalities with the failure characteristics of conventional concrete short beams, they exhibit significant differences and unique features in detail.

1. Elastic Stage: During initial loading, the steel skeleton, reinforcing bars, and concrete should exhibit small strains while working in concert, with sectional strain varying linearly. Recycled concrete is prone to cracking, but the crack pattern resembles that of ordinary concrete. Due to its smaller shear span-to-depth ratio, TSRSB-3 (λ = 0.76) first develops diagonal cracks followed by bending cracks. The truss-type steel skeleton enhances the cracking load capacity owing to its well-defined load transfer pathways.
2. Elastic-plastic stage: As the load increases, cracks proliferate. Bending cracks propagate toward the center of the truss-like steel skeleton, while diagonal cracks extend toward the loading point and supports. The specimen beam operates under crack conditions.
3. Failure Stage: When the load reached 80% of the peak load, the diagonal crack widened, and concrete spalling occurred in the shear span section. The diagonal crack width exceeded the maximum crack width limit of 0.3 mm specified in China’s Standards^34^ for structural members under Class I environmental conditions. The specimen failed due to shear diagonal compression. TSRSB-5 (λ = 1.52) exhibited shear bond failure due to its larger shear span-to-depth ratio. The truss-type steel skeleton enables the short beam to maintain substantial load-bearing capacity even after concrete spalling, resulting in a higher load compared to an equivalent reinforced concrete beam without the truss-type steel skeleton.

### Load-displacement curve

In this test, the load-displacement curve serves as a key parameter for evaluating shear performance. By testing specimens under varying shear span-to-depth ratios and recycled coarse aggregate replacement rates, the following critical data and insights were obtained.

#### The effect of the shear span-to-depth ratio

**Fig. 12:**
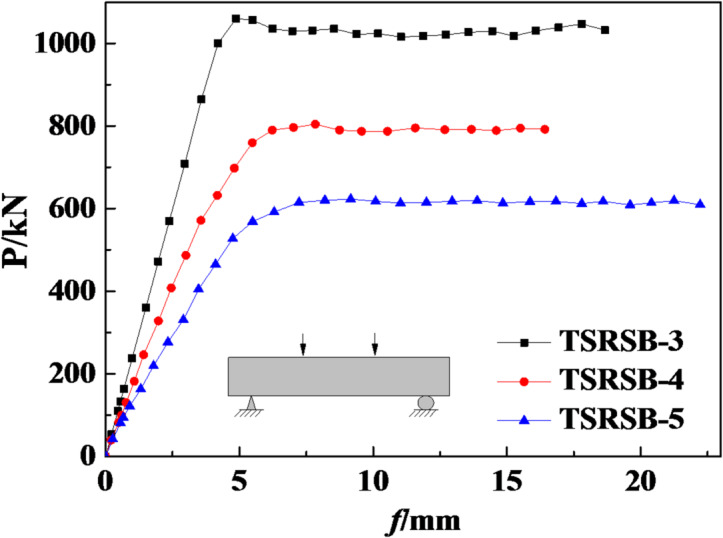
Load-mid-span deflection curve of different shear span-to-depth ratios.

When investigating the shear performance of TSRSBs, the shear span-to-depth ratio is a key factor whose effect is significant. The data presented in Fig. 12 reveals a distinct trend: as the shear span-to-depth ratio progressively decreases (from 1.52 to 0.76), both the initial stiffness and ultimate load capacity of the specimens demonstrate significant enhancement. This indicates that specimens with smaller shear span-to-depth ratios exhibit enhanced load-carrying capacity, thus demonstrating the significant effect of this ratio on the shear performance of TSRSBs. Simultaneously, after reaching the ultimate load, the load-carrying capacity of the specimens changes relatively steadily. This is attributed to the excellent deformation capacity of the steel skeleton, enabling the components to exhibit outstanding ductility and energy dissipation properties. Comparing the data in Table 5, using the specimen TSRSB-5 with a shear span-to-depth ratio of 1.52 as the baseline, specimens with reduced shear span-to-depth ratios of 1.14 and 0.76 exhibited ultimate load increases of 29.1% and 70.1%, respectively. This result indicates that reducing the shear span-to-depth ratio effectively enhances the ultimate load capacity of TSRSBs, thereby improving their shear performance.

**Table 5:** Ultimate load of TSRSB of different shear-span ratio.

Specimen number	Shear span-to-depth ratio λ	Ultimate load *P*_u_/kN	Increase rate (%)
TSRSB-3	0.76	1060.0	70.1
TSRSB-4	1.14	804.0	29.1
TSRSB-5	1.52	623.0	-

#### The effect of RCA replacement rate

**Fig. 13:**
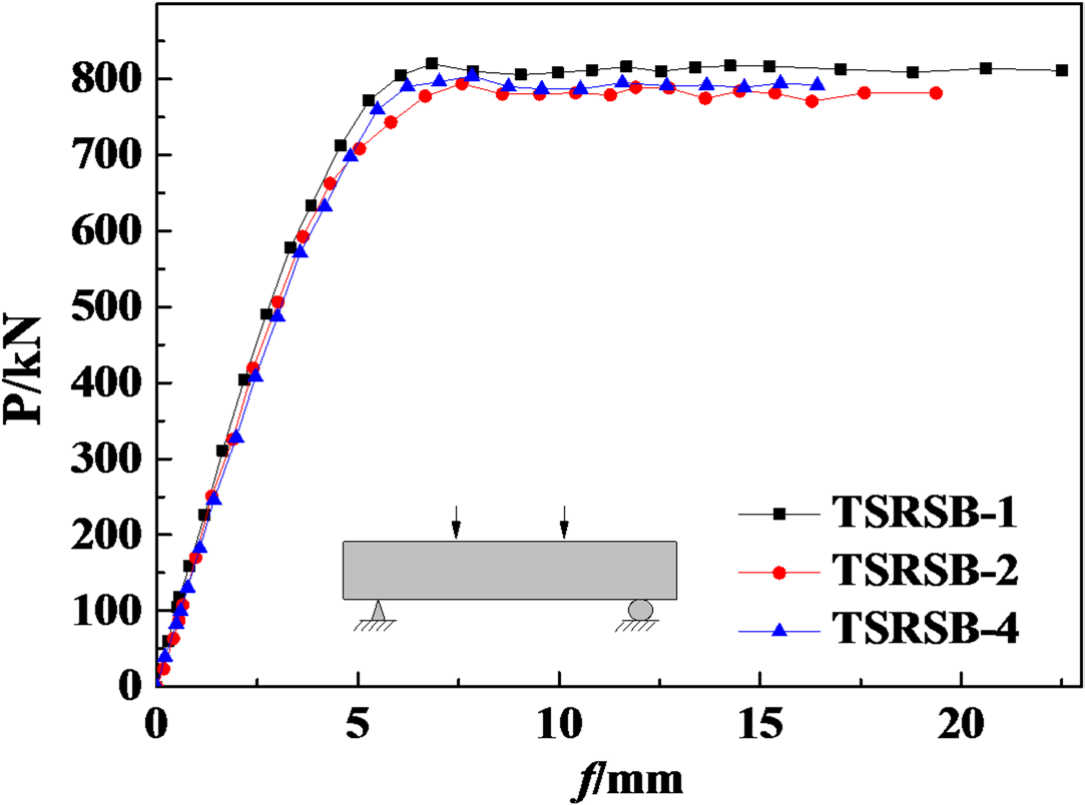
Load-mid-span deflection curve of different RCA replacement rate.

When investigating the application effectiveness of recycled coarse aggregate in specimens, its replacement rate appears to have no significant impact on the specimens’ shear performance. As shown in Fig. 13, when the replacement rate gradually increased from 0% to 100%, the initial stiffness of the specimens did not exhibit significant variation, while the ultimate load showed a trend of first slightly decreasing and then slightly increasing. This behavior indicates that the replacement rate of recycled coarse aggregate has a limited impact on the mechanical properties of the specimens to a certain extent. A more detailed understanding of this trend can be gained through an in-depth analysis of the data in Table 6. Using the truss-shaped steel ordinary concrete short beam (specimen TSRSB-1) as a reference, when the replacement rate of recycled coarse aggregate reached 50% and 100%, the ultimate load of the specimens decreased by only 3.2% and 2.0% compared to the reference value. This minor change further confirms that the replacement rate of recycled coarse aggregate has a relatively limited impact on the ultimate load of truss-shaped specimens.

**Table 6:** Ultimate load of TSRSB of different RCA replacement rate.

Specimen number	Shear span-to-depth ratio λ	Ultimate load *P*_u_/kN	Decline rate (%)
TSRSB-1	0	820.0	-
TSRSB-2	50	794.0	3.2
TSRSB-4	100	804.0	2.0

### Load-strain curve

In the test, a detailed analysis of the load-strain curve is crucial for understanding the load-bearing behavior of short beams. By analyzing the strain in the concrete, the strain in the steel members, and the strain in the stirrups, we gain a deeper understanding of the mechanical properties of TSRSBs under shear loading.

#### Concrete strain analysis

**Fig. 14:**
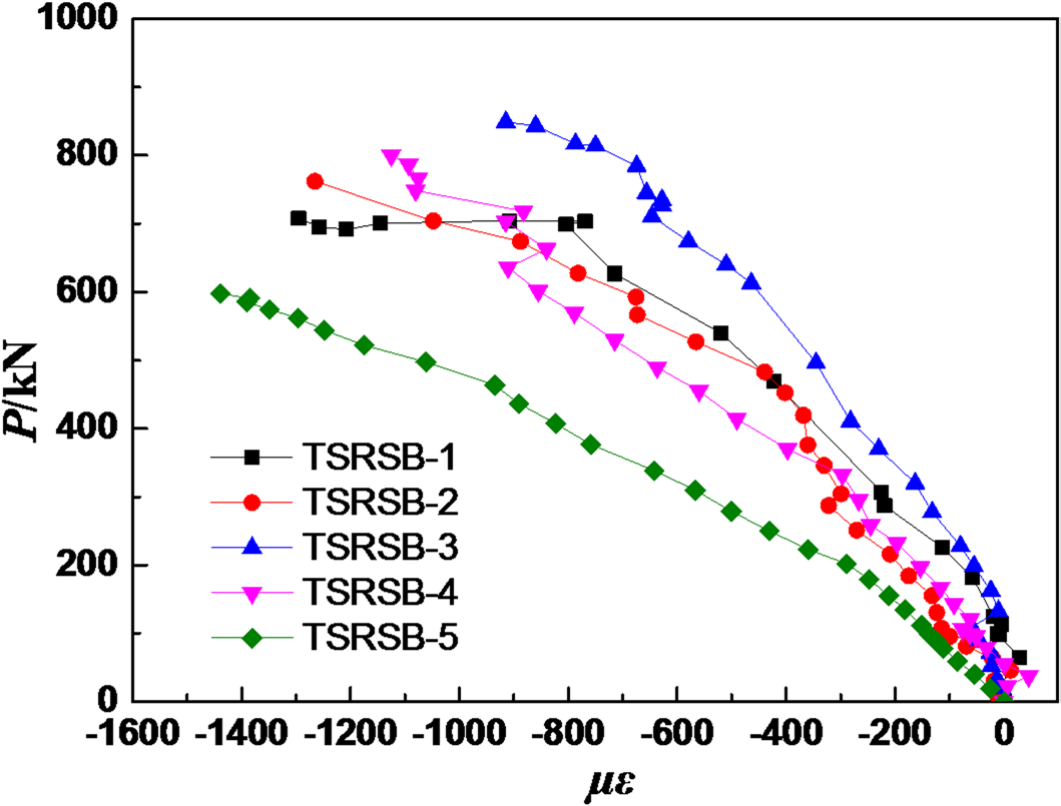
The load-strain curves of concrete oblique compression rod.

**Fig. 15:**
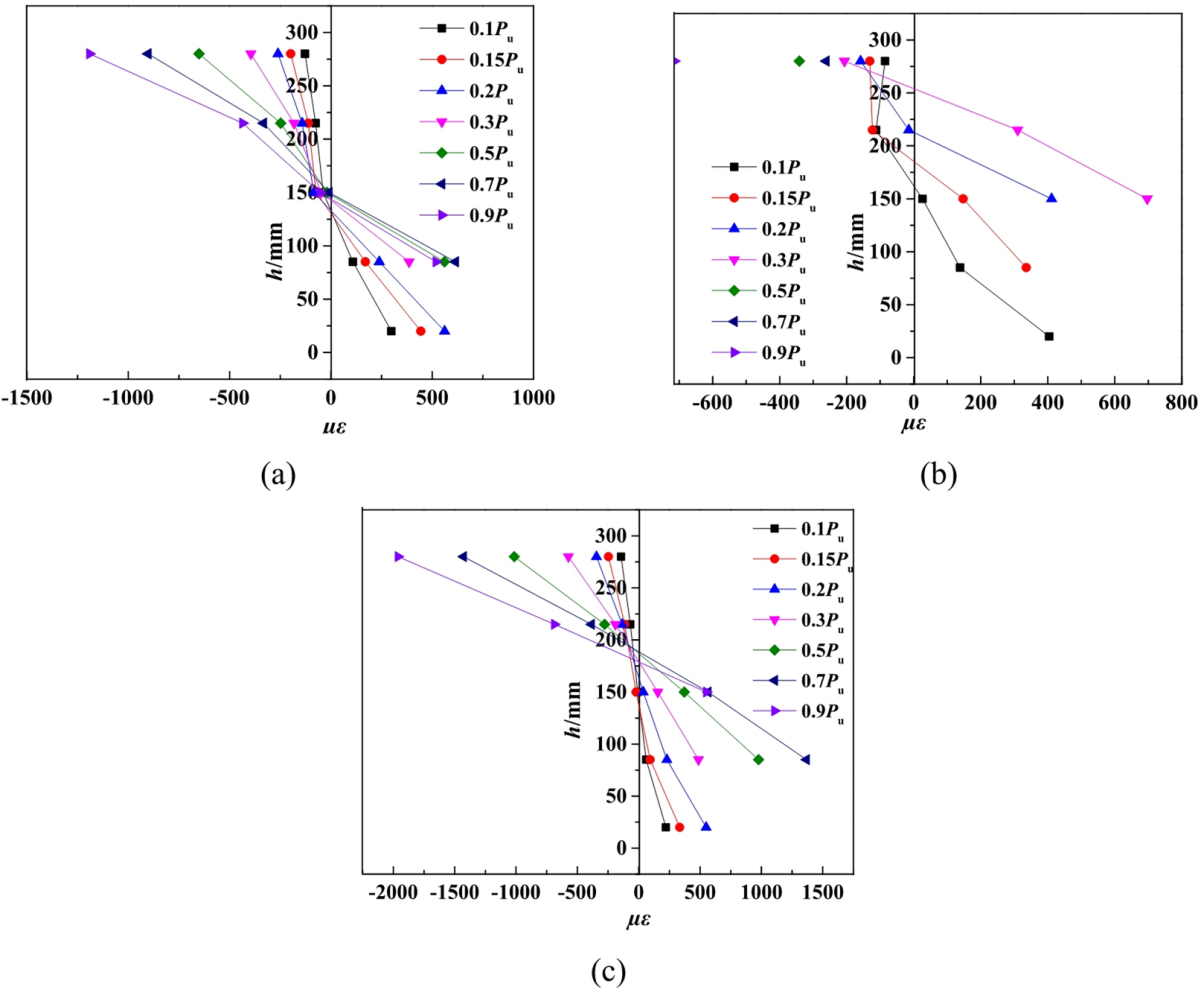
Strain distribution of concrete in specimens mid-span across section. (**a**) TSRSB-3, (**b**) TSRSB-4, (**c**) TSRSB-5.

Through in-depth investigation of concrete’s mechanical behavior, we found that varying shear span-to-depth ratio significantly influences stress distribution in both inclined and frontal sections of the structure. Figure 14 illustrates the load-strain curve for concrete inclined-support structures, revealing that as the shear span-to-depth ratio decreases, the rate of strain increase in inclined-support concrete structures accelerates. This phenomenon highlights the heightened sensitivity of short beams to shear stress under low shear span-to-depth ratios. Additionally, Fig. 15 reveals the concrete stress distribution at the mid-span section of the frame-type test specimen. Notably, the concrete strain analysis highlights the influence of the shear span-to-depth ratio on both the diagonal and frontal section strains. Figure 14 presents the load-concrete diagonal compression member strain curve for the shear span section. Diagonal section strain analysis indicates that as the shear span-to-depth ratio decreases, the rate of increase in concrete strain within the diagonal compression member accelerates, demonstrating that short beams exhibit heightened sensitivity to shear forces at lower shear span-to-depth ratios. Figure 15 shows the concrete strain distribution at the mid-span section of the truss specimen. Analysis of the normal section strain reveals nonlinear characteristics in the concrete strain distribution at the mid-span section, particularly under low shear span-to-depth ratios. The formation of diagonal cracks creates a unique mechanical structure. This structure functions as a truss-arch system, with the tensioned longitudinal reinforcement and the tensioned flanges of the T-shaped steel serving as tension members, the beam top as a compression member, the shear span section as a diagonal compression member, and the stirrups and vertical web members acting as vertical tension members. This configuration results in a low-stress zone in the lower mid-span region, where the concrete stress distribution in both compression and tension zones progressively deviates from the conventional assumption of a uniform cross-section.

#### Shaped steel strain curve

**Fig. 16:**
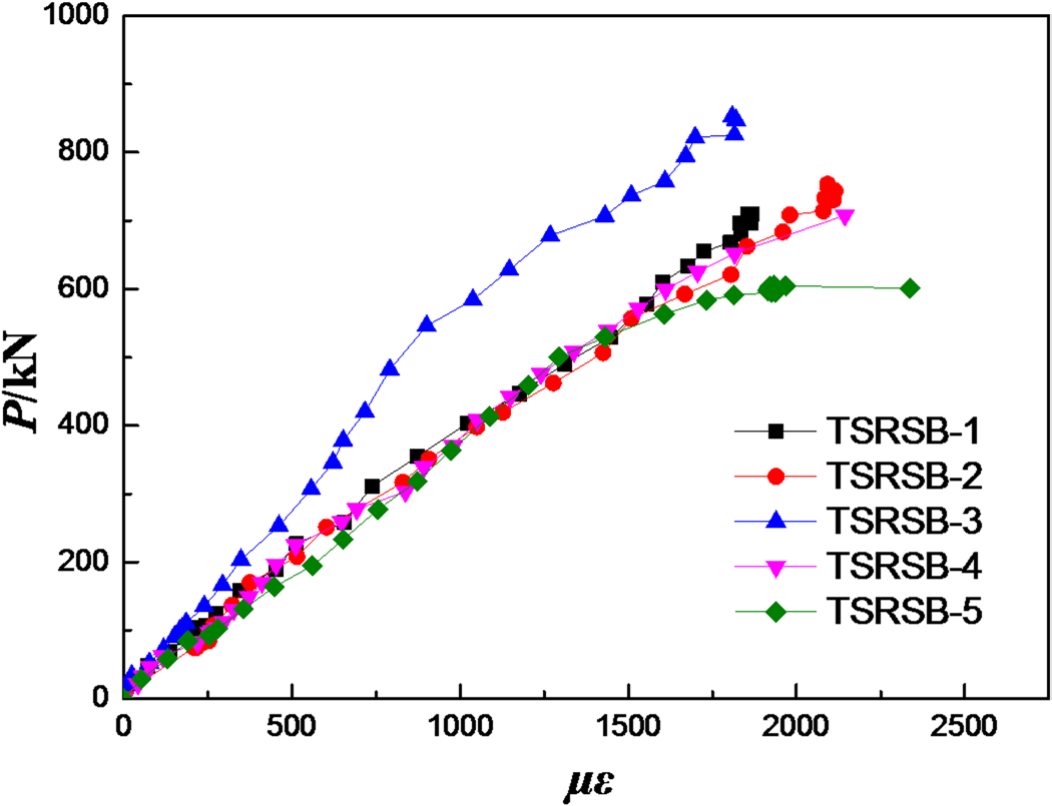
The load-strain curves of steel lower flange.

Figure 16 presents the relationship curve between load and lower flange strain at the mid-span section of the truss-type specimen. As the load approaches its maximum capacity, the deformation in the tensile zone of the lower flange becomes progressively more pronounced with increasing shear span-to-depth ratio. This change indicates that the shear span-to-depth ratio exerts a significant influence on the internal stress distribution and stress state of the structural steel.

#### Stirrup strain curve

**Fig. 17:**
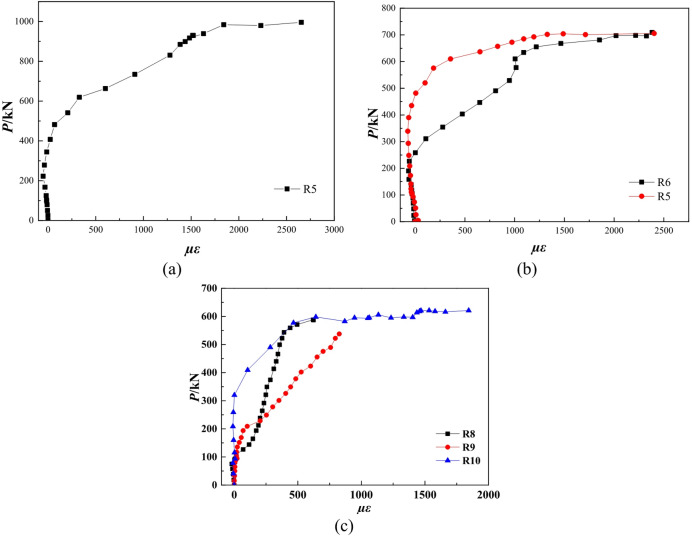
The load-strain curves of stirrups. (**a**) TSRSB-3, (**b**) TSRSB-4, (**c**) TSRSB-5.

Figure 17 illustrates the load distribution and stirrup strain distribution in the shear span section of the truss specimen under concentrated loading conditions. Through in-depth analysis of stirrup strain, specific data regarding the stress behavior of the shear span section can be obtained. At each stage of the test, precise strain measurement points R5, R6, R7, R8, R9, and R10 were established on the stirrups within the specimen’s shear span region. It should be noted that the number of stirrups within the shear span differs among specimens due to the varying shear span lengths and the fixed stirrup spacing of 100 mm (1, 2, and 3 stirrups for TSRSB-3, -4, and − 5, respectively). Figure 17 presents the most representative strain data for each specimen. Refer to the specimen strain distribution diagram detailed in Fig. 5 for the precise locations of these points. During the stage where cracks had not yet appeared in the concrete diagonal compression members, the stirrups and concrete worked together to bear the compressive load. However, as loading continued, significant variations in stirrup strain emerged at different locations within the shear span section, primarily influenced by the shear span-to-depth ratio. Specifically, when the specimen’s shear span-to-depth ratio reached 1.52 (as observed in specimen TSRSB-5), the first location exhibiting significant strain increase was the mid-span stirrups within the shear span region. In contrast, for another specimen with a shear span-to-depth ratio of 1.14 (e.g., TSRSB-4), the first noticeable change in stirrup strain occurred near the loading point. This phenomenon reveals the critical influence of the shear span-to-depth ratio on stress distribution within specimens: at a shear span-to-depth ratio of 1.52, the mid-span region of the shear span zone becomes the area of highest stress concentration. Conversely, when the shear span-to-depth ratio is reduced to 1.14, the shortened shear span zone necessitates only two stirrups. Consequently, the stirrup near the loading point experiences a more unfavorable stress distribution.

## Finite element analysis

### Finite element model establishment

#### Material constitutive

For the material properties of TSRSBs, this model employs two primary material constitutive relationships: steel and recycled concrete (RAC).

(1) Steel.

**Fig. 18:**
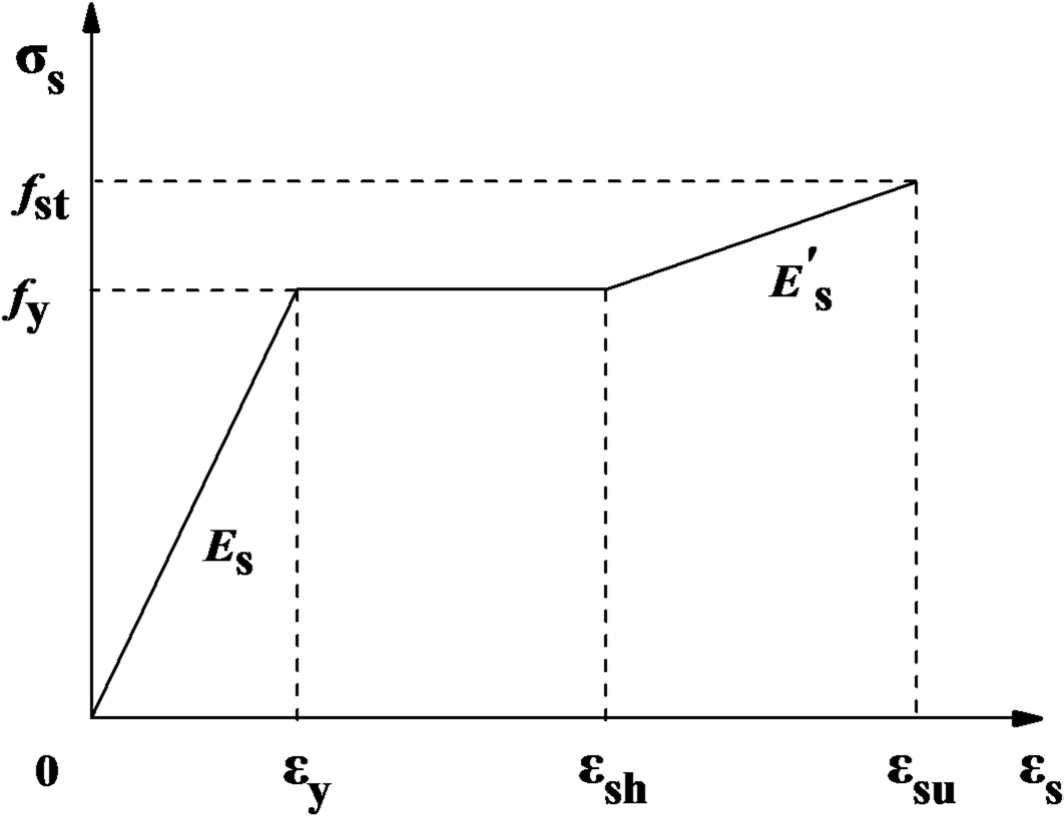
The constitutive relation of steel.

In ABAQUS finite element simulations, the constitutive relationship of steel is modeled using a three-piece spline, as shown in Fig. 18, to comprehensively reflect its elastic, yield, and hardening stages.

(2) RAC.

The constitutive relationship for RAC accounts for the influence of its replacement rate on concrete strength and strain, employing a corresponding mathematical model. In this study, for the compressive RAC constitutive relationship in beams, the model referenced in the literature^35^ was adopted but significantly improved. Building upon Guo’s classical concrete constitutive model^36^, it considers the impact of recycled coarse aggregate replacement rate on concrete performance. The improved constitutive equation is presented as follows:

1 $$y=\left\{ {\begin{array}{*{20}{c}} {ax+(3 - 2a){x^2}+(a - 2){x^3},}&{0 \leqslant x<1} \\ {\frac{x}{{b{{(x - 1)}^2}+x}},}&{x \geqslant 1} \end{array}} \right.$$

Where, $x=\frac{\varepsilon }{{{\varepsilon _{\mathrm{c},\mathrm{r}}}}}$,$y=\frac{\sigma }{{f_{{\mathrm{c},\mathrm{r}}}^{{}}}}$, *r* denotes the replacement rate of recycled coarse aggregate, ${f_{\mathrm{c},\mathrm{r}}}$ denotes the compressive strength of concrete with the measured replacement rate of recycled coarse aggregate; ${\varepsilon _{\mathrm{c},\mathrm{r}}}$ denotes the peak strain corresponding to $f_{{\mathrm{c},\mathrm{r}}}^{{}}$; *a* and *b* are the parameters influencing the replacement rate of recycled coarse aggregate, whose expressions are given by Eqs. (2) and (3):

2 $$a=2.2(0.748{r^2} - 1.231r+0.975)$$

3 $$b=0.8(7.6483r+1.142)$$

The constitutive relationship for the tensile RAC in this paper adopts the constitutive model specified in the Code for Design of Concrete Structures, with the constitutive equation as follows:

4 $$y=\left\{ {\begin{array}{*{20}{c}} {1.2x - 0.2{x^6},}&{0 \leqslant x<1} \\ {\frac{x}{{0.31{f_{t,r}}^{2}{{(x - 1)}^{1.7}}+x}},}&{x \geqslant 1} \end{array}} \right.$$

Where, $x=\frac{\varepsilon }{{{\varepsilon _{\mathrm{t},\mathrm{r}}}}}$, $y=\frac{{{\sigma _{}}}}{{{f_{\mathrm{t},\mathrm{r}}}}}$, *r* denotes the replacement rate of recycled coarse aggregate, $f_{{\mathrm{t},\mathrm{r}}}^{{}}$ denotes the measured tensile strength of concrete with recycled coarse aggregate replacement ratio, ${\varepsilon _{\mathrm{t},\mathrm{r}}}$ is the peak strain corresponding to $f_{{\mathrm{t},\mathrm{r}}}^{{}}$.

When selecting an appropriate concrete damage model, the ABAQUS software library offers diverse options, including the concrete diffuse cracking model, the concrete plastic damage model, and the concrete brittle cracking model. Through in-depth comparative studies and validation through practical application testing, the concrete plastic damage model has been determined to exhibit excellent accuracy and convergence performance when simulating concrete behavior under static loading. The finite element model was precisely developed according to the actual dimensions of the structural members. For the recycled aggregate concrete, the Concrete Damaged Plasticity (CDP) model in ABAQUS was adopted. The key parameters were set as follows: a dilation angle of 30°, an eccentricity of 0.1, and default values for the ratio of biaxial to uniaxial compressive yield stress (*f*_b0_/*f*_c0_ = 1.16) and the second stress invariant parameter (*K*_c_ = 0.667). A viscosity parameter of 0.0005 was included to enhance convergence in the implicit analysis. These parameter selections align with established modeling practices for concrete, as referenced in Wang et al.^37^. Furthermore, a Poisson’s ratio of 0.2 was assigned to the concrete, consistent with reported values for recycled aggregate concrete at high replacement ratios^38^. Therefore, we selected this model as the foundation for this study. For the concrete compression damage factor *d*_c_, we propose the following calculation method:

5 $${d_c}=1 - \frac{{{\sigma _c}E_{0}^{{ - 1}}}}{{\varepsilon _{c}^{{pl}}(1/{b_c} - 1)+{\sigma _c}E_{0}^{{ - 1}}}}$$

Where, ${\sigma _\mathrm{c}}$ is the compressive stress of concrete, *E*_0_ is the initial elastic stiffness of concrete, ${b_\mathrm{c}}=\varepsilon _{\mathrm{c}}^{{\mathrm{p}\mathrm{l}}}/\varepsilon _{\mathrm{c}}^{{\mathrm{i}\mathrm{n}}}$, $\varepsilon _{\mathrm{c}}^{{\mathrm{p}\mathrm{l}}}$ is the plastic strain of concrete under compression, and the nonlinear elastic strain of concrete is $\varepsilon _{\mathrm{c}}^{{\mathrm{i}\mathrm{n}}}$, where $\varepsilon _{\mathrm{c}}^{{\mathrm{i}\mathrm{n}}}={\varepsilon _\mathrm{c}}-\varepsilon _{\mathrm{c}}^{{\mathrm{e}\mathrm{l}}}={\varepsilon _\mathrm{c}}-{\sigma _\mathrm{c}}/{E_0}$。.

The tensile damage factor *d*_t_ for concrete is expressed as follows:

6 $${d_{\mathrm{t}}}=1 - \frac{{{\sigma _{\mathrm{t}}}E_{0}^{{ - 1}}}}{{\varepsilon _{{\mathrm{t}}}^{{{\mathrm{pl}}}}(1/{b_{\mathrm{t}}} - 1)+{\sigma _{\mathrm{t}}}E_{0}^{{ - 1}}}}$$

Where, ${\sigma _\mathrm{t}}$ is the tensile stress in concrete, ${b_\mathrm{t}}=\varepsilon _{\mathrm{t}}^{{\mathrm{p}\mathrm{l}}}/\varepsilon _{\mathrm{t}}^{{\mathrm{i}\mathrm{n}}}$, $\varepsilon _{\mathrm{t}}^{{\mathrm{p}\mathrm{l}}}$ is the plastic strain of concrete under tension, and the nonlinear elastic strain of concrete is $\varepsilon _{\mathrm{t}}^{{\mathrm{i}\mathrm{n}}}$, where $\varepsilon _{{\mathrm{t}}}^{{{\mathrm{in}}}}={\varepsilon _{\mathrm{t}}} - \varepsilon _{{\mathrm{t}}}^{{{\mathrm{el}}}}={\varepsilon _{\mathrm{t}}} - {\sigma _{\mathrm{t}}}/{E_0}$.

#### Interactions and boundary conditions

In the model, the spacers and RAC beams are connected using a “bound” constraint to simulate their state of no relative motion. The interactions between the structural steel, structural steel skeleton, and reinforcing steel skeleton with the RAC are assumed to be fully bonded, with no slip considered. The perfect-bond assumption is appropriate for specimens without significant slip (TSRSB-1 to TSRSB-4). For TSRSB-5, the model can approximate the ascending branch and peak load but cannot simulate the post-peak slip-induced failure.

#### Load application and element selection

**Fig. 19:**
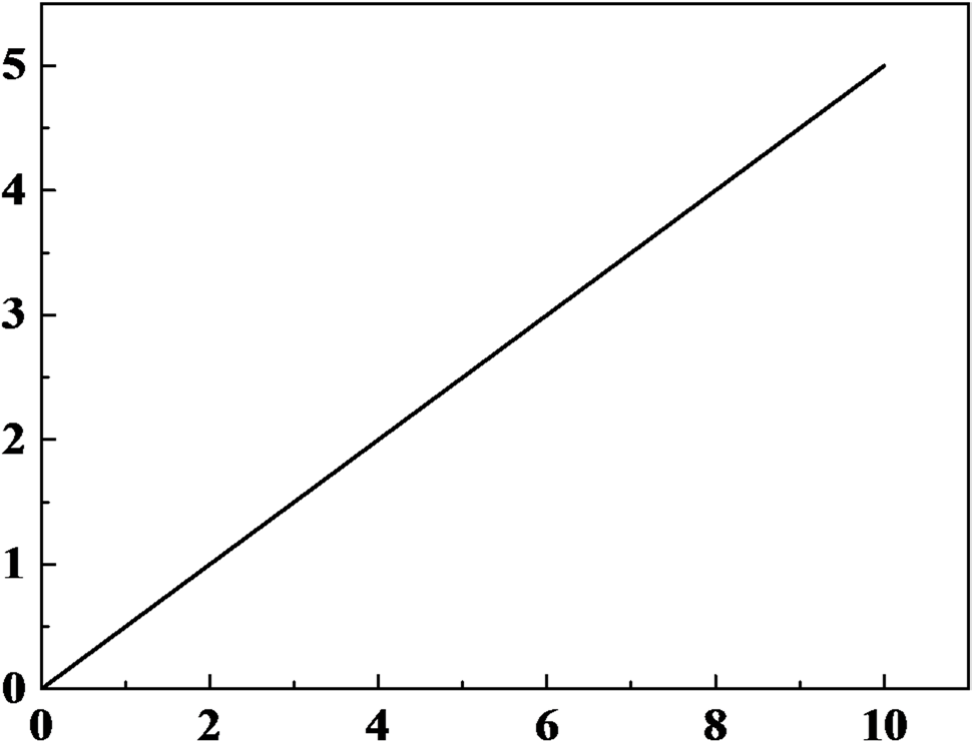
Curve of loading amplitude.

This study comprises two analysis steps. First, boundary constraints were applied at the beam ends. Then, in the initial analysis step, displacement-controlled loading was applied following the loading amplitude curve shown in Fig. 19 to simulate the loading process during the experiment. Regarding element selection, C3D8R solid elements were used for the RAC beam and pads, C3D8I solid elements for the steel sections and steel skeleton, and T3D2 truss elements for the reinforcement skeleton.

#### Mesh generation and solving

**Fig. 20:**
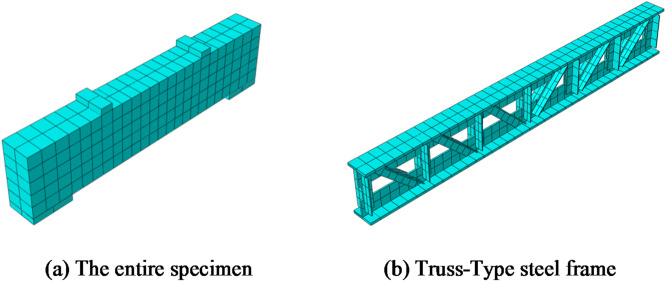
The mesh of model.

In finite element analysis, the mesh refinement strategy plays a crucial role. For this study, a customized meshing approach was implemented based on the specific properties of each component and the required computational accuracy. Specifically, the base mesh size for the upper block and RAC beam was set to 60 mm, while the lower block utilized a base size of 75 mm. For solid-web steel sections, a base size of 25 mm was selected, whereas truss-type steel frames employed a base size of 35 mm. Following this meticulous meshing process, rigorous quality checks were performed to ensure the mesh met subsequent analysis requirements. The mesh layout for the specimen in the finite element simulation is detailed in Fig. 20. Using the analysis system, an analysis task was successfully generated for the established model data, and the job was submitted without issue. During computation, if anomalies such as non-convergence were encountered, the model data underwent comprehensive and meticulous inspection, with corresponding adjustments made based on actual conditions. Upon completion of all calculations, advanced visualization tools were employed to conduct an in-depth and comprehensive post-processing analysis of the obtained results.

### Finite element model validation

In this study, finite element model validation serves as a critical step to ensure the accuracy and reliability of simulation results. Using ABAQUS, a powerful finite element analysis tool, a comprehensive and in-depth simulation study was conducted on the shear performance of the truss-shaped steel recycled concrete short beam (Model: TSRSB-4). By comparing simulated and measured data on displacement, stress distribution, load-deflection response, and ultimate load-carrying capacity, the accuracy and reliability of this finite element model in simulating the shear behavior of the TSRSB were validated.

**Fig. 21:**
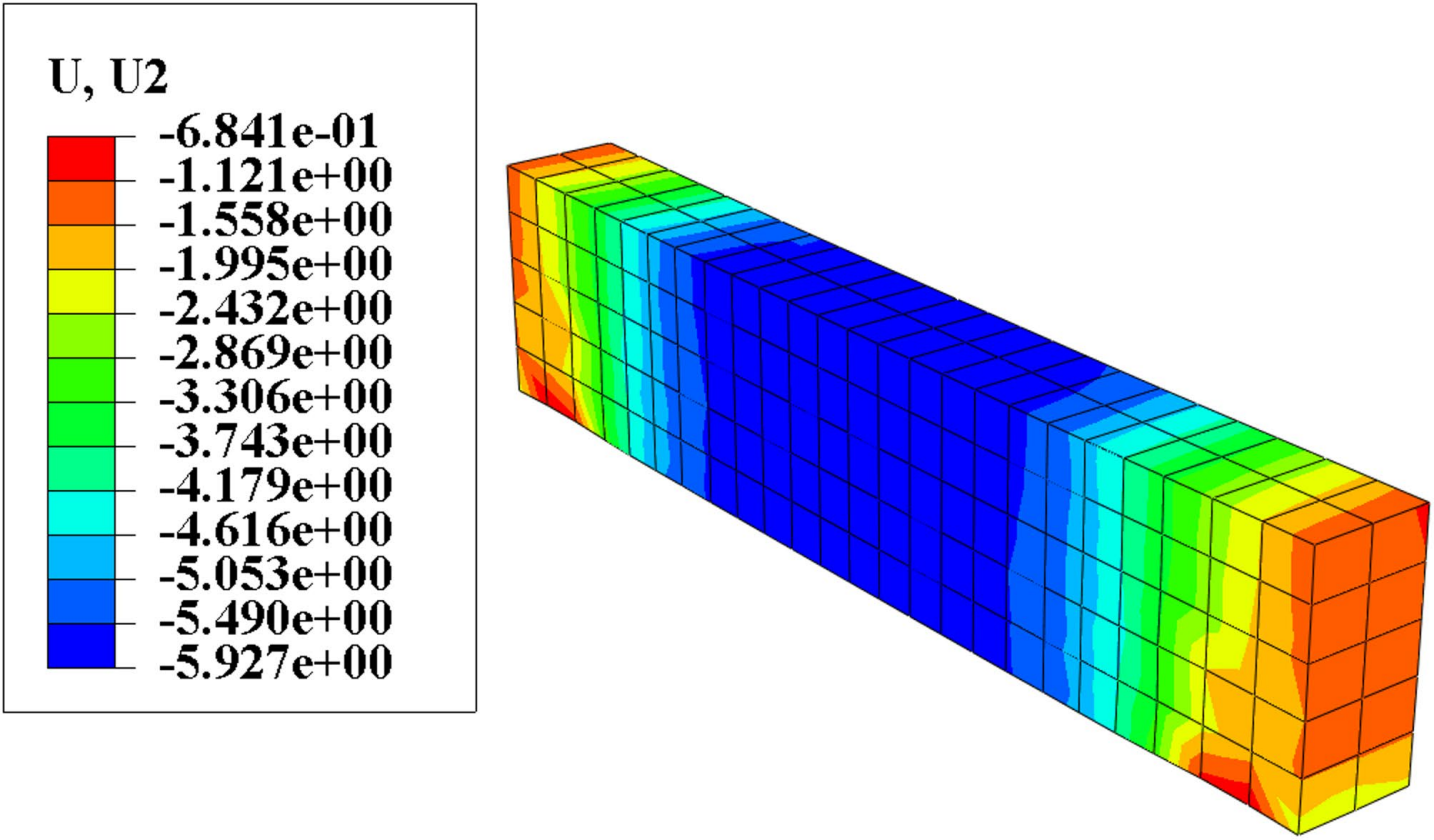
Vertical displacement cloud of specimens.

**Fig. 22:**
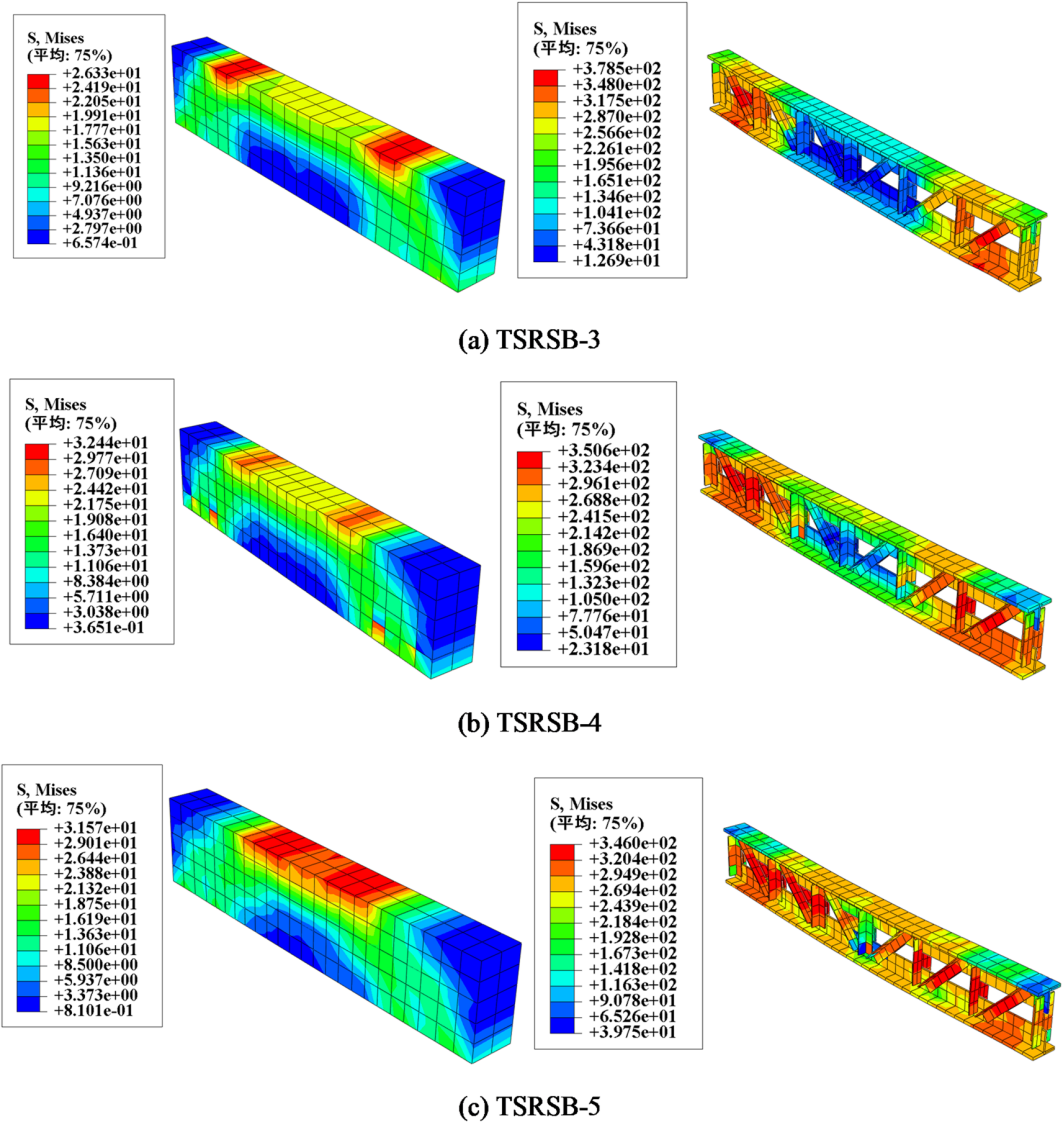
Stress cloud of specimens.

The vertical displacement simulation under ultimate load (as shown in Fig. 21) reveals a symmetrical displacement distribution centered on the mid-span section, which closely matches the displacement pattern observed under actual loading conditions. Simulation results indicate a mid-span deflection of 5.927 mm, which closely matches actual test data. Further analysis of stress distributions in concrete and steel reinforcement (as shown in Fig. 22) reveals that maximum stresses gradually shift toward the beam ends as the shear span-to-depth ratio decreases, consistent with observed phenomena. Additionally, the simulation accurately captured stress concentration in the upper spacer block region and areas of elevated stress along the line connecting the concrete loading point to the support. This further validates the model’s accuracy in simulating stress distribution.

**Fig. 23:**
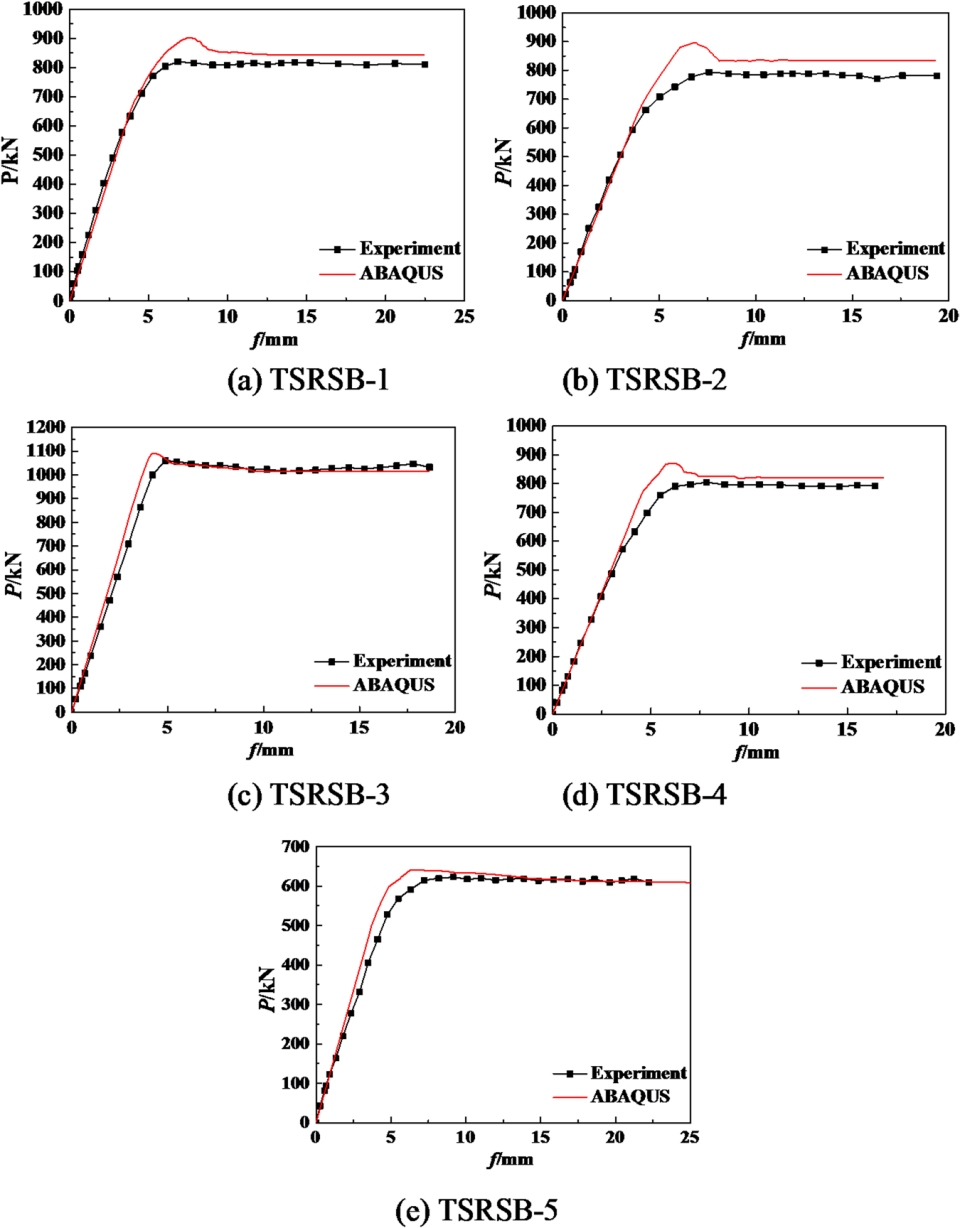
Comparison of specimen’s load-deflection curves.

When comparing the load-deflection curves of the test specimens (as shown in Fig. 23), it was observed that during the initial loading phase, the simulated curve closely matched the experimental curve. As the load increased, although some discrepancies emerged between the two, their overall trends remained consistent. These discrepancies are primarily due to minor variations in specimen fabrication and curing processes, as well as the perfect-bond assumption adopted in the model, which cannot capture the bond-slip behavior observed in some specimens (e.g., TSRSB-5). Nevertheless, the simulation still provides a reasonable approximation of the ascending branch and peak load. Finally, we compared the simulated ultimate load values with the experimental values for the truss-type specimens (as shown in Table 7). The results indicate that the ratio of simulated to experimental values ranged from 1.03 to 1.13, with an average of 1.07 (average error of 7%), further validating the accuracy of the finite element model in predicting the ultimate load-bearing capacity of the specimens.

**Table 7:** Comparison of simulated load and measured load.

Specimen number	Ultimate load *P*_u_/kN		*P*_u, m_ /*P*_u, e_
	Simulated value *P*_u, m_	Test value *P*_u, e_	
TSRSB-1	902.5	820	1.10
TSRSB-2	895.9	794	1.13
TSRSB-3	1090	1060	1.03
TSRSB-4	869.4	804	1.08
TSRSB-5	641	623	1.03

Note: The mean value of the ratio between simulated and test values (Pu, m /Pu, e) is *µ* = 1.07, with a standard deviation *σ* = 0.04 and a coefficient of variation of *C*v = 0.04.

### Finite element stress analysis

In the field of civil engineering, recycled concrete is gaining widespread attention as an environmentally friendly and economical building material. However, its performance is influenced by multiple factors, and research on its load-bearing capacity is particularly crucial when combined with structural steel. This paper focuses on the shear performance of TSRSBs through finite element stress analysis. It investigates the influence of factors such as recycled coarse aggregate replacement rate, shear span-to-depth ratio, and beam configuration on their load-bearing capacity. Using ABAQUS finite element software, a numerical model of the steel-reinforced recycled concrete short beam was established. The model’s validity was verified through comparison with experimental results. Building upon this foundation, a multi-parameter extended analysis was conducted to compensate for the limitations of experimental testing.

#### The effect of the shear span-to-depth ratio

Based on the TSRSB-4 model, four specific shear span-to-depth ratios (0.76, 1.14, 1.52, 1.90) were selected for detailed finite element simulation expansion studies. These four distinct shear span-to-depth ratios correspond to four test specimens, designated as TSRSB4-0.76, TSRSB4-1.14, TSRSB4-1.52, and TSRSB4-1.90. Throughout the simulation process, all other parameters remained constant except for the shear-to-span ratio.

**Fig. 24:**
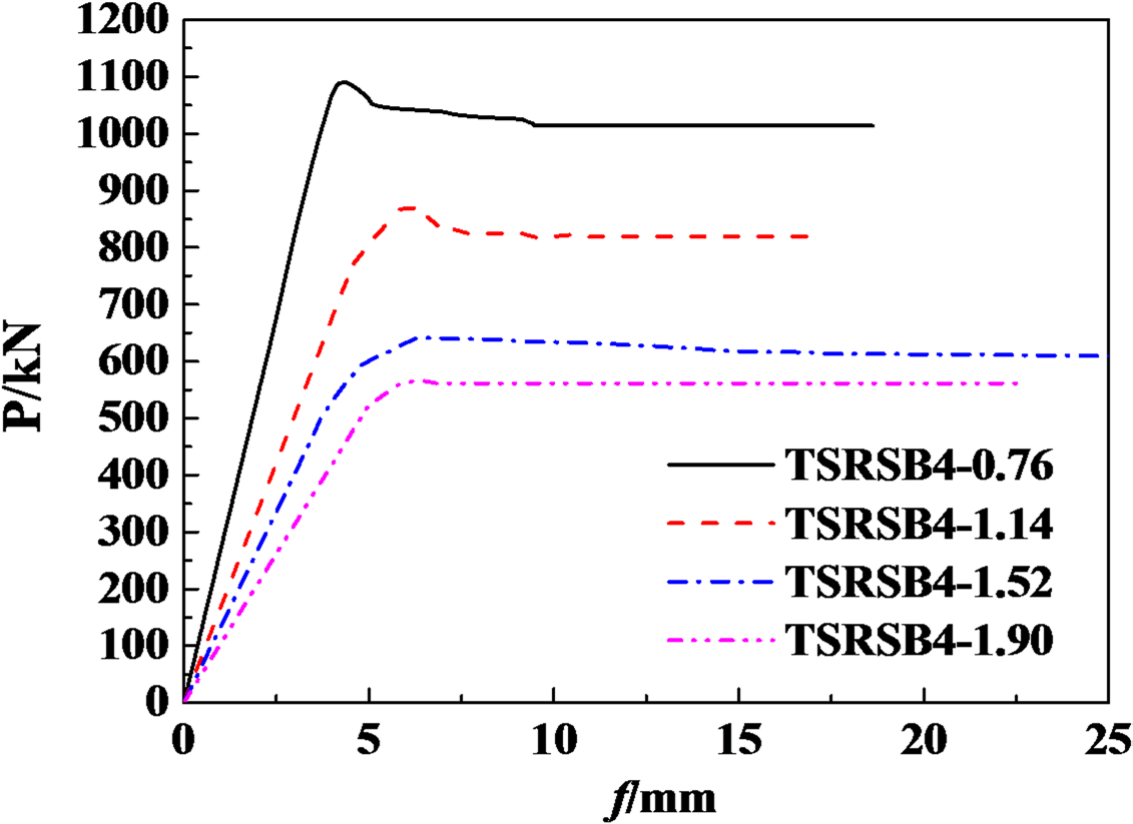
Load-mid-span deflection curve at different shear span-to-depth ratios.

Through in-depth finite element analysis, load-deflection diagrams encompassing various shear span-to-depth ratio factors were constructed, as shown in Fig. 24. This diagram visually illustrates how varying shear span-to-depth ratios influence specimen performance. The load-deflection curve clearly demonstrates that the shear-to-span ratio significantly modulates the initial stiffness of specimens during the elastic stage. As the shear span-to-depth ratio progressively decreases, the specimens exhibit a marked trend toward enhanced ultimate load-carrying capacity (Table 8).

**Table 8:** Ultimate load.

Specimen number	Shear span-to-depth ratio λ	Ultimate load *P*_u_/kN	Increase rate /%
TSRSB4-0.76	0.76	1090.0	92.7
TSRSB4-1.14	1.14	839.4	48.4
TSRSB4-1.52	1.52	641.0	13.4
TSRSB4-1.90	1.90	565.5	-

#### The effect of RCA replacement rate

**Fig. 25:**
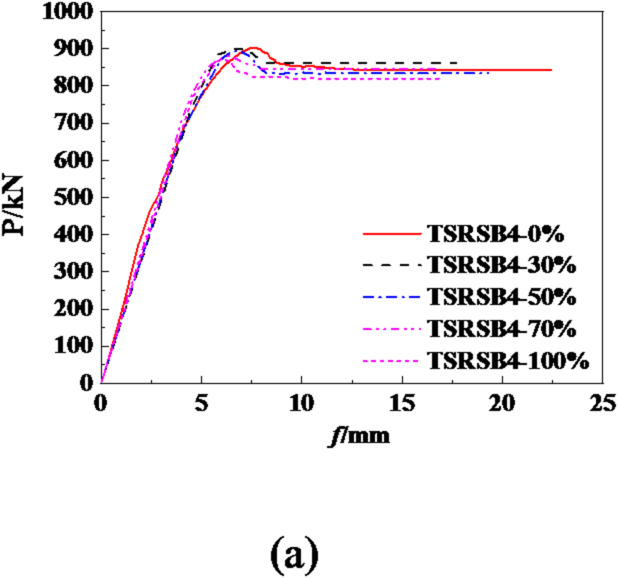
Load-mid-span deflection curve of different RAC replacement rate.

Using TSRSB-4 as the base model, finite element analysis was employed to investigate the effect of varying replacement ratios of recycled coarse aggregate on structural performance. Five different RCA replacement ratios were selected: 0%, 30%, 50%, 70%, and 100%. The corresponding specimen codes are TSRSB4-0%, TSRSB4-30%, TSRSB4-50%, TSRSB4-70%, and TSRSB4-100%. Under conditions where all other parameters remained constant, comprehensive simulation analyses were conducted. The finite element simulation results yielded the load-deflection curve shown in Fig. 25, which visually illustrates the impact of different RCA replacement ratios on specimen performance. Observing the chart data reveals that during the elastic deformation stage of the specimens, the replacement rate of RCA did not significantly affect the initial stiffness. However, it is noteworthy that as the replacement rate gradually increased, although the maximum load borne by the specimens exhibited a certain degree of decline, this downward trend was relatively gradual, with the magnitude of change not being significant.

Referring to the data in Table 9, it can be observed that compared to specimens without recycled concrete (i.e., replacement rate of 0%), the ultimate load of specimens decreased by 0.04%, 0.7%, 2.4%, and 3.7% respectively when the replacement rates reached 30%, 50%, 70%, and 100%. This trend indicates that the ultimate load of specimens gradually decreases as the replacement rate increases. As the replacement rate rises, internal damage within the specimens also increases. However, it is noteworthy that the reduction in load is relatively small. This is primarily because the steel reinforcement not only provides excellent load-bearing capacity but also effectively suppresses the propagation of internal damage within the recycled concrete.

**Table 9:** Ultimate load of different RAC replacement rate.

Specimen number	Replacement rater/%	ultimate load *P*_u_/kN	Decline rate /%
TSRSB4-0%	0	902.5	-
TSRSB4-30%	30	902.1	0.04
TSRSB4-50%	50	895.9	0.7
TSRSB4-70%	70	880.8	2.4
TSRSB4-100%	100	869.4	3.7

#### The effect of beam types

**Fig. 26:**
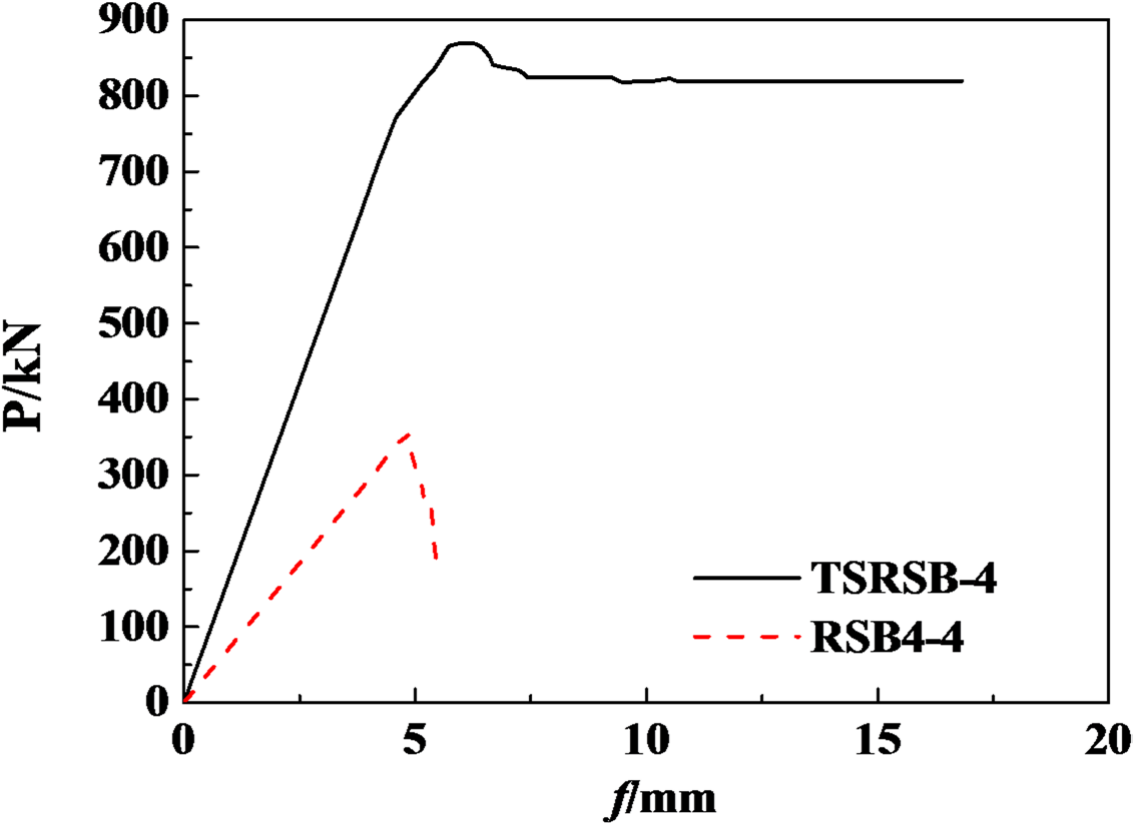
Load-mid-span deflection curve of different beam form.

Based on the TSRSB-4 benchmark design, the structure was optimized while maintaining other technical details constant. The original steel-reinforced configuration was removed, leading to the innovative development of the reinforced steel-recycled concrete short beam (RSB-4). Through high-precision numerical modeling analysis, we successfully obtained comparative data on load-deflection characteristics for both structures, along with their respective ultimate load-carrying capacities, as shown in Fig. 26. The comparison revealed an intriguing phenomenon: although the original TSRSB exhibited higher stiffness during the initial loading stage, the steel-ribbed TSRSB-4 demonstrated superior deformation capacity near the ultimate load. Unlike the RSB-4, it did not rapidly lose load-bearing capacity due to brittle failure. This difference significantly highlights the crucial role of steel ribs in optimizing specimen failure modes and enhancing member ductility.

Further analysis of the data in Table 10 reveals that, using the ultimate load of RSB-4 as a reference, the ultimate load-bearing capacity of TSRSB-4 increased by 144.4%. This figure fully demonstrates the significant effect of steel reinforcement in enhancing the ultimate load-bearing capacity of test specimens.

**Table 10:** Ultimate load of different beam form.

Specimen number	Shape steel types	ultimate load *P*_u_/kN	Increase rate /%
TSRSB-4	Truss-type	869.4	144.4
RSB-4	None	355.7	-

## Shear capacity calculation method

### The current calculation formula for shear bearing capacity

To accurately establish the shear capacity formula for TSRSBs, actual test results were compared with prior research findings. Drawing upon references^22–29^, a shear capacity calculation formula for TSRSBs was developed. This paper does not enumerate all relevant formulas here, focusing instead on the theoretical foundation most closely aligned with this study: the shear capacity calculation method proposed by Deng. Deng^29^ proposed a formula for calculating shear bearing capacity under concentrated load conditions as follows:

7 $${V_\mathrm{u}}=\frac{{0.14}}{\lambda }{f_\mathrm{c}}b{h_0}+0.388\left( {{f_{\mathrm{y}\mathrm{v}1}}\frac{{{A_{\mathrm{s}\mathrm{v}1}}}}{{{s_1}}}{h_0}+{f_{\mathrm{y}\mathrm{v}2}}\frac{{{A_{\mathrm{s}\mathrm{v}2}}}}{{{s_2}}}{h_0}+{f_{\mathrm{y}\mathrm{v}3}}{A_{\mathrm{s}\mathrm{v}3}}\mathrm{s}\mathrm{i}\mathrm{n}\theta } \right)+\frac{{0.586}}{\lambda }{f_\mathrm{s}}{t_\mathrm{w}}{h_\mathrm{w}}$$

Where, *f*_yv1_ is the design strength value of the stirrups; *A*_sv1_ is the total cross-sectional area of all legs of stirrups within the same cross-section; ${s_1}$ is the spacing of stirrups along the length of the specimen; *f*_yv2_ is the design strength value of vertical web members;*A*_sv2_ is the total cross-sectional area of all legs of vertical web members within the same cross-section; ${s_2}$ is the spacing of vertical web members along the specimen length; *f*_yv3_ denotes the design value of the diagonal web member strength; *A*_sv3_ denotes the total cross-sectional area of all legs of the diagonal web member within the same cross-section; *θ* denotes the angle between the diagonal web member and the beam axis; *f*_s_ denotes the design value of the tensile strength of the steel section web.

**Table 11:** Comparison of calculated and experimental values.

Specimen number	f_cu_ / MPa	Replacement rate *r* / %	shear span-to-depth ratio λ	V_u,cal_	V_u,exp_	V_u,cal_ /V_u,exp_
TSRSB-1	43.3	0	1.14	361.8	410.0	0.88
TSRSB-2	39.5	50	1.14	344.3	397.0	0.87
TSRSB-3	41.2	100	0.76	465.7	530.0	0.88
TSRSB-4	41.2	100	1.14	350.1	402.0	0.87
TSRSB-5	41.2	100	1.52	292.4	311.5	0.94

Note: The mean value of the ratio between simulated and test values (P_u, m_ /P_u, e_) is *µ* = 0.89, with a standard deviation *σ* = 0.03, and a coefficient of variation of *C*_v_=0.03.

The calculated and experimental shear capacities are compared in Table 11. The formula proposed by Deng et al. yields a mean ratio of calculated-to-experimental shear capacity (*V*_u, cal_/*V*_u, exp_) of 0.89, with a standard deviation of 0.03. It is noted that a mean value below 1.0 indicates that Deng’s formula provides, on average, a conservative estimate of the shear capacity. This conservatism is a fundamental and often desirable characteristic for design-oriented formulas, as it incorporates a safety margin to account for material variability and uncertainties, aligning with standard structural engineering practice.

### Theoretical analysis of shear capacity

In current academic research, regarding the calculation of shear capacity in the diagonal sections of truss-type steel-concrete composite beams, scholars both domestically and internationally generally focus on two mainstream calculation methods:

1. Calculate the shear capacity of the T-shaped steel as a solid web section. The shear capacity of the vertical web members and diagonal web members shall be determined based on the shear resistance of the stirrups, and the sum of this capacity and the shear capacity of the concrete shall constitute the shear capacity of the specimen.
2. Calculate the shear capacity of the steel skeleton and reinforced concrete separately, then combine them to determine the overall shear capacity of the specimen.

**Fig. 27:**
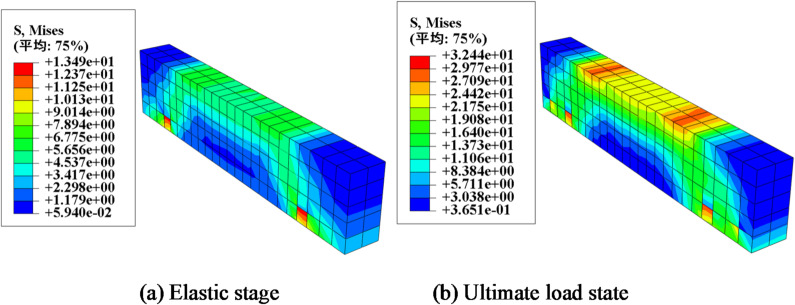
Stress evolution course of typical concrete.

**Fig. 28:**
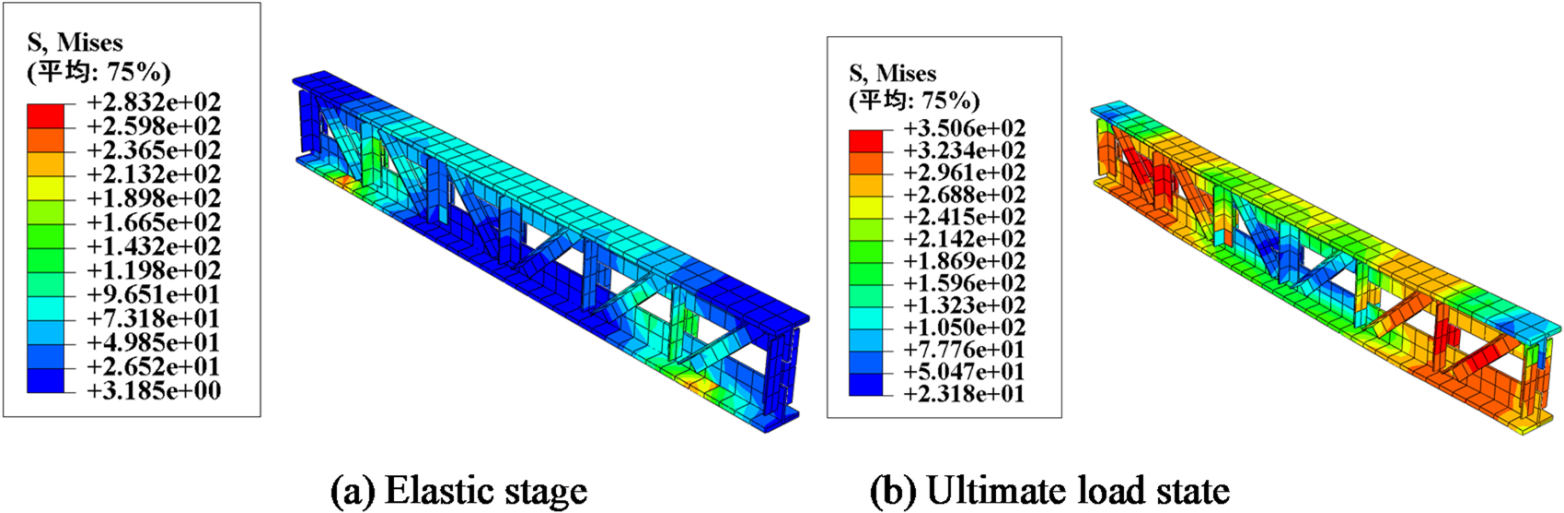
Stress evolution course of typical steel reinforced.

In the detailed analysis of the concrete-steel composite structures shown in Figs. 27 and 28, the characteristics of stress distribution evolution across stages were carefully examined, and the force transfer mechanisms were observed. The stress distribution diagrams reveal that high stress concentrations primarily occur in the compression zone formed by the concrete and steel web plates. This zone plays a crucial role in force transmission, effectively transferring diagonal compressive forces to the supports. Simultaneously, the top of the beam primarily experiences compressive forces, while the bottom primarily bears tensile forces through the tension flanges of the steel sections and longitudinal reinforcement. Additionally, the vertical web members function as vertical tie rods within the structure. These elements collectively form the key components of the truss-arch model, including diagonal compression members, upper chord compression members, lower chord tension members, and vertical tie rods. Comparing stress distribution diagrams between the elastic and ultimate load stages reveals pronounced stress concentrations near the loading points and support connections in the shear span section during initial loading. This reflects efficient force transfer through diagonal compression members to the support structure. Upon reaching ultimate load, the force transmission path becomes more distinct, with pronounced stress concentration occurring at the supports. Although low-stress zones in the midspan decrease, extensive stress distribution persists overall. This aligns with the observed progressive development of compressive deformation and bending cracks at the loading point during testing.

**Fig. 29:**
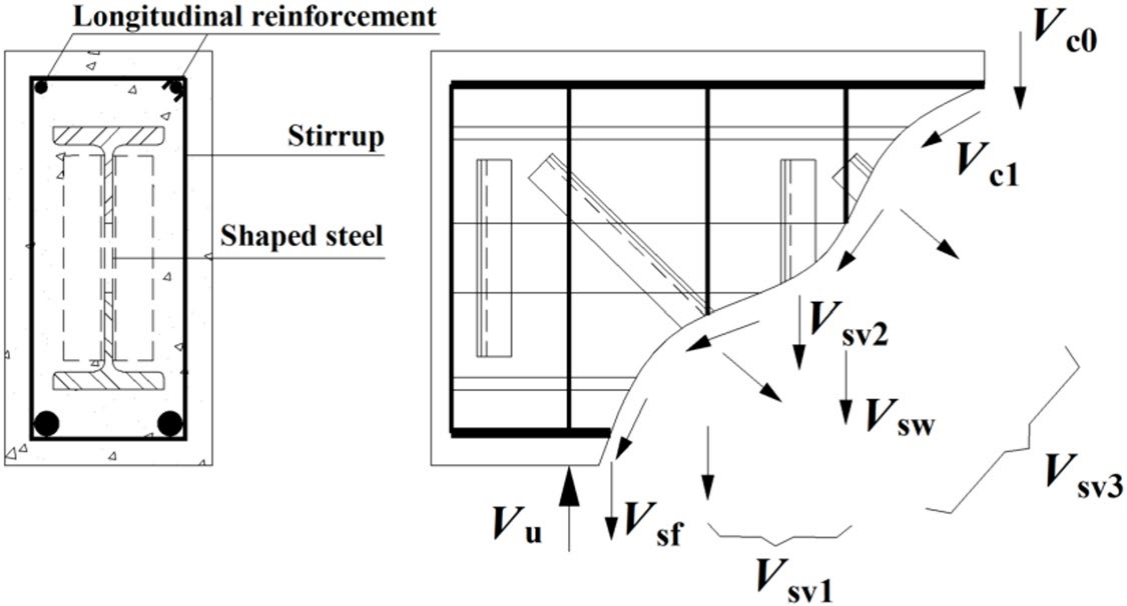
Theoretical model.

Based on experimental research and incorporating domestic and international shear capacity calculation methods, this paper comprehensively considers the failure mode of truss-type short beams, the replacement rate of recycled concrete, and the truss-arch stress model. The resulting theoretical stress model is shown in Fig. 29. The shear capacity formula for TSRSBs can be uniformly expressed as:

This study conducted an in-depth investigation into the failure modes of truss-shaped steel short beams based on a series of detailed experiments and advanced methods in shear capacity calculation from both domestic and international sources. Throughout this process, particular attention was given to the replacement ratio of recycled concrete and the influence of the truss-arch stress model. Based on these comprehensive considerations, a unified stress theory model was successfully developed, as illustrated in Fig. 29. This model provides a unified calculation formula (8) for the shear capacity of TSRSBs.

8 $${V_\mathrm{u}}={V_\mathrm{c}}+{V_{sv}}+{V_{\mathrm{s}\mathrm{w}}}$$

Where: *V*_c_ is the shear capacity of concrete, i.e., *V*_c_= *V*_c0_ +*V*_c1_ +*V*_sf_ ; *V*_sv_ is the shear capacity of stirrups and web members, i.e., *V*_sv_ =*V*_sv1_ +*V*_sv2_ +*V*_sv3_; *V*_sv1_ is the shear capacity of the stirrups, *V*_sv2_ is the shear capacity of the vertical diagonal members, and *V*_sv3_ is the shear capacity of the inclined diagonal members; *V*_sw_ is the shear capacity of the steel sections.

The proposed truss-arch model relies on a critical localized interaction between the internal steel truss and the concrete diagonal strut. The diagonal steel web members, oriented along the principal tensile stress path, serve a dual function: (1) they control and segment the diagonal cracking in the concrete, thereby defining the boundaries and enhancing the stability of the ensuing concrete compression strut; and (2) they actively participate as tensile ties in the truss mechanism after cracking. This physical interference redirects the internal force flow: the transverse tensile forces that would otherwise need to be sustained by the concrete’s tensile strength and aggregate interlock are instead carried directly by the yielding of the diagonal steel webs. This stress redistribution allows the concrete to primarily resist diagonal compression, leading to the observed compressive strut failure mode. The vertical web members and stirrups provide necessary confinement to this composite strut, completing the interactive truss-arch action.

### Formula for calculating the shear capacity of TSRSBs

In this paper, based on the in-depth analysis and discussion presented earlier, a formula for the shear capacity of recycled concrete short beams with truss-shaped steel members has been derived, specifically addressing the complex characteristics of their shear performance. During the development of this practical calculation formula, the following three key assumptions were made to ensure the formula’s accuracy and applicability.

1. The shear resistance of the T-beam flanges is not considered;
2. The shear capacity of TSRSBs is determined by the combined shear resistance of the recycled concrete, T-shaped steel web, vertical web members, diagonal web members, and stirrups;
3. The effect of bond slip between truss steel and recycled concrete on the shear capacity of short beams is accounted for through three correction factors: recycled concrete, T-beam web, and stirrup web. These correction factors are determined based on regression analysis of experimental data and relevant domestic and international research findings.

Based on domestic and international research findings regarding the shear capacity of truss-shaped steel-concrete composite shallow beams under concentrated loads, and incorporating actual data obtained from this experiment, the influence of recycled coarse aggregate replacement rate and shear span-to-depth ratio on performance was comprehensively considered. On this basis, a multiple regression mathematical model Eq. (9) was established to accurately describe the shear capacity characteristics of TSRSBs. This model provides a powerful tool for deepening the understanding and predicting the performance of this type of beam in actual engineering applications.

9 $${V_\mathrm{u}}=\alpha \frac{{{A_1}}}{{\lambda +{A_2}}}{f_t}b{h_0}+{A_3}\left( {{f_{\mathrm{y}\mathrm{v}1}}\frac{{{A_{\mathrm{s}\mathrm{v}1}}}}{{{s_1}}}{h_0}+{f_{\mathrm{y}\mathrm{v}2}}\frac{{{A_{\mathrm{s}\mathrm{v}2}}}}{{{s_2}}}{h_0}+{f_{\mathrm{y}\mathrm{v}3}}\frac{{{A_{\mathrm{s}\mathrm{v}3}}}}{{{s_3}}}{h_0}\mathrm{s}\mathrm{i}\mathrm{n}\theta } \right)+\frac{{{A_4}}}{\lambda }{f_\mathrm{s}}{t_\mathrm{w}}{h_\mathrm{w}}$$

Where: A_1_, A_2_, A_3_, A_4_ are undetermined coefficients.

Through regression analysis of experimental data, the following formula for calculating the shear capacity of TSRSBs under concentrated loading can be derived:

10 $${V_\mathrm{u}}=\alpha \frac{{1.87}}{{\lambda - 0.04}}{f_t}b{h_0}+0.39\left( {{f_{\mathrm{y}\mathrm{v}1}}\frac{{{A_{\mathrm{s}\mathrm{v}1}}}}{{{s_1}}}{h_0}+{f_{\mathrm{y}\mathrm{v}2}}\frac{{{A_{\mathrm{s}\mathrm{v}2}}}}{{{s_2}}}{h_0}+{f_{\mathrm{y}\mathrm{v}3}}\frac{{{A_{\mathrm{s}\mathrm{v}3}}}}{{{s_3}}}{h_0}\mathrm{s}\mathrm{i}\mathrm{n}\theta } \right)+\frac{{0.58}}{\lambda }{f_\mathrm{a}}{t_\mathrm{w}}{h_\mathrm{w}}$$

Where: The numerical coefficients (1.87, -0.04, 0.39, 0.58) are the determined values for A1, A2, A3, A4, obtained from regression analysis of the experimental data; *α* is the influence coefficient of recycled coarse aggregate replacement rate, taken as *α* = 20/(20 + *r*);${s_3}$denotes the spacing of diagonal web members along the specimen’s length.

The reduction factor α is introduced to account for the diminished efficiency of the recycled concrete diagonal strut within the composite ‘truss-arch’ mechanism. It reflects the degradation of the concrete’s contribution (*V*c) due to the increased porosity, weakened interfacial transition zones, and lower fracture energy of RAC, which impair its ability to sustain diagonal compression and provide aggregate interlock.

**Table 12:** Comparison of calculated and experimental values.

Specimen number	f_cu_ / MPa	Replacement rate *r* / %	shear span-to-depth ratio λ	V_u,c_	V_u,e_	V_u,c_ /V_u,e_
TSRSB-1	43.3	0	1.14	406.9	410.0	0.99
TSRSB-2	39.5	50	1.14	393.8	397.0	0.99
TSRSB-3	41.2	100	0.76	532.4	530.0	1.00
TSRSB-4	41.2	100	1.14	393.6	402.0	0.98
TSRSB-5	41.2	100	1.52	325.4	311.5	1.04

Note: The mean value of the ratio between simulated and test values (*V*_*u*,c_ /*V*_*u*,e_) is *µ* = 1.00, with a standard deviation *σ* = 0.01 and a coefficient of variation of *C*v = 0.01.

By comparing the calculated values obtained using the formula proposed in this paper with the actual experimental values from Table 12, it was found that the mean ratio between the two remained stable at 1.00, with both the standard deviation and coefficient of variation being only 0.01. This result fully validates the accuracy of the formula constructed in this paper. This formula not only thoroughly considers the impact of recycled concrete replacement ratio and shear span-to-depth ratio on structural performance but also incorporates necessary adjustments through carefully designed correction factors. It fills a gap in the field of calculating the shear capacity of TSRSBs, with errors controlled within 5%. Owing to the rational steel reinforcement design and clear force transmission pathways, the calculation precision is enhanced. These results indicate that, within the tested ranges (*r* = 0-100%,*λ* = 0.52–1.14), the proposed formula provides a reasonable fit to the experimental data. However, due to the limited sample size (five specimens), the formula should be considered as an empirical approximation specific to this study; further validation with independent experimental data is required before it can be used for general prediction. Because the regression is based on only five specimens, the formula should be considered as an empirical approximation valid only within the tested ranges (*r* = 0-100%,*λ* = 0.52–1.14). Its extrapolation beyond these ranges or use as a general design equation is not recommended without further validation. Should it be adapted for direct use in design, appropriate safety or resistance factors would need to be calibrated and applied to cover a wider range of material and geometric uncertainties, following established code formats.

## Conclusions

This paper presents a comprehensive investigation into the shear behavior of a novel Truss-Type Steel-Reinforced Recycled Concrete Short Beam (TSRSB) through experimental testing and finite element analysis. The study systematically examines the effects of key parameters, including the replacement ratio of RCA (*r*) and the shear span-to-depth ratio (*λ*), on the failure mode, load-bearing capacity, and deformation characteristics. A sophisticated finite element model was developed and validated against experimental results, demonstrating high accuracy in predicting the structural response. Furthermore, a practical mechanical model and calculation formula for the shear capacity of TSRSB were proposed, which accurately account for the contributions of recycled concrete, the truss-type steel skeleton (including vertical and diagonal web members), and stirrups. Based on the experimental and analytical results, the following major conclusions can be drawn:

1. The failure mode of TSRSBs is significantly influenced by the shear span-to-depth ratio (*λ*). For short beams with smaller *λ* values (0.76 and 1.14), the primary failure mode is shear-compression failure, characterized by the formation of parallel diagonal struts between the loading point and the support. In contrast, the beam with a larger *λ* (1.52) exhibited shear-bond failure, associated with noticeable slip between the steel and concrete.
2. Reducing the shear span-to-depth ratio dramatically improves the shear capacity and initial stiffness of TSRSB. The ultimate load of the specimen with *λ* = 0.76 was 70.1% higher than that of the specimen with *λ* = 1.52, highlighting the profound “short beam effect” and the efficient arching action developed in deep beams.
3. Within the 0% -100% range studied, the influence of the RCA replacement ratio on the shear capacity was not significant. The maximum reduction in ultimate load was only 3.2%, indicating that the presence of the robust truss-type steel skeleton effectively compensates for the potential weaknesses of recycled concrete, making TSRSB a viable and sustainable structural element.
4. The stress distribution observed from finite element analysis confirms that the shear resistance mechanism of TSRSB can be effectively idealized as a combined “truss-arch” model. In this model, the diagonal concrete struts, the top concrete chord, the bottom steel tension chord, and the vertical/diagonal steel web members work synergistically to transfer shear forces.
5. A newly developed calculation formula for the shear capacity of TSRSB is proposed. This formula incorporates a reduction factor (*α* = 20/(20 + *r*)) to account for the recycled aggregate’s influence and accurately sums the contributions of various components.

## Data Availability

The original contributions presented in this study are included in the article. Further inquiries can be directed to the corresponding author.

## Abbreviations

b: Width of beam cross-section
h: Total height of beam cross-section
h_0_: Effective depth of beam cross-section
h_w_: Height of steel web
t_w_: Thickness of steel web
a: Shear span length
λ: Shear span-to-depth ratio (λ = a / h_0_)
s: Spacing of stirrups
s_1_: Spacing of stirrups along the beam length
s_2_: Spacing of vertical web members along the beam length
s_3_: Spacing of diagonal web members along the beam length
θ: Angle between diagonal web member and beam axis
f_y_: Yield strength of steel
f_u_: Ultimate strength of steel
f_yv1_: Yield strength of stirrups
f_yv2_: Design strength of vertical web members
f_yv3_: Design strength of diagonal web members
f_c_: Axial compressive strength of concrete
f_cu_: Cube compressive strength of concrete
f_c,r_: Axial compressive strength of concrete with a specific RCA replacement ratio
f_t_: Axial tensile strength of concrete
f_t,r_: Axial tensile strength of concrete with a specific RCA replacement ratio
E_s_: Elastic modulus of steel
E_c_: Elastic modulus of concrete
f: Mid-span deflection
r: Replacement ratio of recycled coarse aggregate (%)
α: Influence coefficient for RCA replacement ratio (α = 20/(20 + r))
ε: Strain
ε_c,r_: Peak compressive strain of concrete corresponding to f_c, r_
ε_t,r_: Peak tensile strain of concrete corresponding to f_t, r_
d_c_: Compressive damage factor of concrete
d_t_: Tensile damage factor of concrete
V: Shear force
V_u_: Shear capacity
V_u,cal_: Calculated shear capacity
V_u,exp_: Experimental shear capacity
V_c_: Shear capacity contributed by concrete
V_sv_: Shear capacity contributed by web members and stirrups
V_sw_: Shear capacity contributed by steel web
P_u_: Ultimate load
P_u,m_: Simulated ultimate load
P_u,e_: Experimental ultimate load
A_sv_: Cross-sectional area of steel bar or web member
A_sv1_: Cross-sectional area of stirrups
A_sv2_: Cross-sectional area of vertical web members
A_sv3_: Cross-sectional area of diagonal web members
µ: Mean value
σ: Standard deviation
C_v_: Coefficient of variation
